# Development and psychometric evaluation of the Oneness Experience Scale

**DOI:** 10.3389/fpsyg.2026.1789746

**Published:** 2026-08-03

**Authors:** Eric Van Lente, Michael J. Hogan

**Affiliations:** School of Psychology, University of Galway, Galway, Ireland

**Keywords:** consciousness measurement, ego dissolution, factor analysis, non-dual awareness, oneness experience, psychometric scale development, self-transcendence, well-being

## Abstract

**Introduction:**

Experiencing oneness or nondual awareness has long been considered a core aspect of spirituality and an aim of meditation and spiritual practice. Oneness experience may lead to well-being and has various adaptive functions in everyday life. However, there has been a lack of comprehensive measures of oneness experiences that are grounded in the consensus-based reports of experienced meditation practitioners. The purpose of this study is to develop a new measure of oneness experience that is psychometrically robust and suitable for research on mindfulness, spirituality, and well-being.

**Methods:**

Scale items were developed based on prior collective intelligence research and a comprehensive literature review and were subsequently reviewed by five content experts. The dimensionality of the scale was examined using exploratory factor analysis (EFA) and confirmatory factor analysis (CFA) in a large sample of participants (*N* = 762; age: *M* = 37.29, SD = 17.16, Female = 66%).

**Results:**

Results revealed an 84-item scale with 21 factors, including: *Part of a Whole/Connection With Everything*, *Ego-dissolution*, *Clarity*, *Nowness*, *Safety and Reassurance*, *Non-judgementality*, *Timelessness*, *Spontaneity*, *Peace*, *Compassion*, *Self-compassion*, *Non-striving*, *Spaciousness*, *Acceptance*, *Non-separation from Others*, *Revelation Of Truth*, *Flow*, *Perfect As It Is*, *Openness*, *Appreciation*, and *Same and Different*. The scale and its subscales possess good internal consistency and construct validity. Evidence of construct validity was found through positive associations with well-being (as measured by the Mental Health Continuum), mindfulness (using the Five Facet Mindfulness Questionnaire), personality (based on the Big Five Inventory-10), and self-reports of oneness experience.

**Discussion:**

*The Oneness Experience Scale* is a measure suited for process- and outcomes-based research in the domains of mindfulness, spiritual practices, and well-being.

## Introduction

The experience of oneness, of being at one with others and the universe, is frequently reported by people across cultures, both within and outside of religious contexts (Ivanhoe et al., 2018; Van Lente and Hogan, 2020). Oneness experiences can include alterations in the sense of self such as feeling unseparated from everything or a feeling of the self dissolving (Yaden, Haidt et al., 2017). These experiences have been described across a range of traditional oneness-related concepts such as Nirvikalpa samadhi, Buddha nature, non-dual awareness, Theosis, and Henosis (Taylor and Egeto-Szabo, 2017).

There is a long history of inquiry in relation to oneness experiences and a growing body of research in the field of psychology. James (1902/2002, p. 324), in his classic text, *The* Var*ieties of Religious Experience*, highlighted oneness as a core feature of mystical experiences across a variety of traditions including Hinduism, Neoplatonism, Sufism, and Christianity, noting that, “In mystic states we both become one with the Absolute and we become aware of our oneness.*”* Subsequently, Stace (1960, pp. 79, 110) highlighted feelings of *unity* as a feature of mystical experiences and in an effort to measure these experiences, Hood (1975) developed the Mysticism Scale, which includes a *unifying quality* factor which relates to oneness.

More recently, Taylor (2017) highlights oneness as a metacharacteristic of wakefulness, while Newberg and Waldman (2017) propose a *sense of unity or connectedness* as a central component of enlightenment experience. Focusing on the process through which oneness experiences arise, Mills et al. (2018) note that oneness (non-dual awareness) is often regarded as the “ultimate aim” (p. 2) of most types of meditation practice, regardless of traditions.

The idea that oneness is the ultimate aim of meditation practice is consistent with another seminal idea, specifically, that oneness is a fundamental cause of well-being (Ivanhoe et al., 2018). Indeed, oneness experiences are of interest to researchers in part because oneness has been hypothesised to be related to wellbeing, and there is emerging evidence to support this claim. For example, Hood et al. (2009) reported a positive association between mystical experiences and well-being. Mills et al. (2018) found that non-dual awareness (also described as oneness) was associated with lower anxiety and depression. Furthermore, in the context of an intervention study, Dambrun et al. (2019) found that a measure of unified consciousness (including oneness indicators) mediated the positive effect of meditation practice on happiness.

However, our understanding of the relationship between oneness experiences and well-being is limited, in part because there are limited options when it comes to the measurement of oneness experiences. A detailed review of 11 oneness-focused scales (Van Lente, 2025) reveals several categories of limitation. Some scales measure beliefs about oneness rather than the experience itself (e.g., Garfield et al., 2014; Diebels and Leary, 2019). Others focus on the effects of oneness, such as persisting positive changes in attitude and behaviour, rather than on the experience of oneness (e.g., the Enlightenment Scale; Boyd-Wilson and Walkey, 2015). Some scales are composed of items drawn from other instruments that were not themselves designed to measure oneness or nondual awareness (e.g., the Nondual Awareness Dimensional Assessment; Hanley et al., 2018). In other cases, the method used to develop the scale items is unclear (e.g., the Nondual Embodiment Thematic Inventory; Mills et al., 2018). Where scales were grounded in experiential sources, these sources were often limited — for example, the Enlightenment Scale items were developed largely based on the first author’s experience and study, and the Oneness Beliefs Scale items (Garfield et al., 2014) were derived from the writings of a single teacher (Ikeda, 1982). More generally, where existing scales drew on theoretical frameworks [e.g., the Enlightenment Scale; Boyd-Wilson and Walkey (2015); the Nondual Awareness Dimensional Assessment; Hanley et al. (2018)], these were often rooted in conceptions from specific contemplative traditions rather than from scientific or psychological theory grounded in the reports of a diverse group of experiencers.

Two further instruments warrant specific discussion. The Mystical Experience Questionnaire (MEQ; Barrett et al., 2015; MacLean et al., 2012) was designed to capture acute mystical experiences, typically single episodes often assessed in the context of psychedelic research, rather than the broader, potentially enduring qualities of oneness experience that the present scale aims to measure. The Ego Dissolution Inventory (EDI; Nour et al., 2016), while relevant, captures only one facet of oneness (i.e., the dissolution of the sense of self).

In light of these limitations, the current study sought to develop a scale that measures the experience of oneness rather than beliefs about it or its effects; that is grounded in the direct, consensus-based reports of a diverse group of experienced meditators using a rigorous collective intelligence methodology; that comprehensively covers the dimensional complexity of oneness experience; and that is suited for measuring oneness as an ongoing experience rather than an acute episodic state.

### Components of oneness

Integrating research on oneness is difficult due to the varied and often vague conceptual definitions of constructs used across studies. Furthermore, definitions can refer to a single experience (state), or they can refer to dispositional (trait) tendencies. Scales are also varied, with most incorporating affective, wellbeing-related and cognitive components, potentially conflating the experience of oneness with its emotional qualities or downstream effects. Relationships between measures of oneness and various outcomes would be expected to differ based on the different constructs and scale items used across studies. Supplementary Table S1 presents an overview of possible conceptual components of oneness and the way in which they align across different theoretical accounts. In the foundational account provided by William James, four characteristics of such experiences are proposed: *Ineffability* – the sense that the experience cannot be adequately described in words; *Noetic* – a state of insight or revelation experienced as authoritative and objective; *Transiency* – these experiences do not endure; and *Passivity* – one’s will is put aside during the experience.

It is important to note that James’ broader category of religious experience encompasses a wide range of phenomena beyond oneness, including experiences of conversion and saintliness, many of which do not involve a sense of unity or dissolution of self–other boundaries. More generally, religious and mystical experiences are highly diverse, including phenomena such as the perceived presence of spiritual or divine entities (Van Eyghen, 2023) and various alterations in the sense of self that do not entail oneness (Taves, 2020). Nevertheless, because oneness has been widely regarded as a core feature of many mystical experiences [e.g., James (1902/2002, p. 324)], the present study focuses specifically on oneness rather than attempting to capture this broader domain.

Stace (1960) added features to those proposed by James, including *blessedness/peace*, *sacredness*, and *paradoxicality*. Stace also differentiated between *extrovertive oneness* (all things are one and feel alive) and *introvertive oneness* (nonspatial/nontemporal pure consciousness). Pahnke (1966) and Pahnke and Richards (1966) similarly created a typology of the mystical state based on the writings of mystics and scholars of these experiences. The typology includes nine components which are very similar to the components proposed by Stace.

More recent conceptualisations share similar features. Newberg and Waldman (2017) explore the nature of temporary and enduring enlightenment. While overlaps with other accounts are evident, their conceptualisation places additional focus on the experience of *non-striving/flow* and points to more enduring effects of enlightenment experiences such as being more *open-minded* and having greater *intensity of experience*. The work of Kilrea et al. (2023), based on interviews with people who had undergone an experience of spiritual awakening and established spiritual teachers, resulted in the Inventory of Secular/Spiritual Wakefulness (WAKE) scale. The feature list of the scale overlaps with earlier accounts (see the row structure in Supplementary Table S1) but also includes additional changes in everyday experience and behaviour, including presentness and authenticity.

The conceptualisations developed by Costeines (2009) and Fire (2011) are based on qualitative studies that focus more specifically on nondual awareness and consciousness. Costeines (2009) conducted a thematic analysis to examine what nondual consciousness means to skilled meditators. Fire (2011) used a qualitative phenomenological method to explore participants’ felt experience (Gendlin, 1962) of nondual consciousness, working with 8 participants who had regular experience of nondual awareness and could voluntarily evoke it. These research-based accounts by Costeines (2009) and Fire (2011) are further complemented by the integrative review and conceptualisation of non-dual experience provided by Josipovic (2021). Again, the features listed overlap with other accounts, but also include some variation and diversity including reports on *trust*, *luminosity/radiance*, and *bliss*.

### Understanding the relationship between oneness and wellbeing

Understanding the relationship between oneness and wellbeing is challenging. In the broader literature on spirituality and oneness experiences, increased wellbeing is often highlighted as having adaptive functions in everyday life (Edinger-Schons, 2019; Hanley et al., 2018; Koenig, 2015; Koburtay et al., 2023; Mancuso and Lorona, 2022; Mosqueiro et al., 2020). This is evident in theory, qualitative and quantitative research at two levels, where it is argued that wellbeing may be (a) a part of spirituality and oneness [see Koenig (2008)] and (b) a consequence of spirituality and oneness.

As can be seen in Supplementary Table S1, many accounts highlight wellbeing (of different types) as part of oneness-related constructs. For example, Josipovic (2021) includes *bliss* as one of the dimensions of nondual awareness, and bliss is also measured in the Nondual Awareness Dimensional Assessment developed by Hanley et al. (2018). Similarly, Newberg and Waldman (2017) and Taylor (2017) highlight *positive affective states*, joy and happiness, and wellbeing as features of enlightenment and wakefulness, respectively. This concurs with earlier accounts by Stace (1960) and Pahnke (1966) who highlighted *deeply felt positive emotions*, *blessedness* and *peace* in their conceptualisations. As such, we might expect to find wellbeing components in any new measure of oneness that is developed using established approaches of scale development that are grounded in the experience and reports of people who have experienced oneness. However, because the inclusion of wellbeing components within conceptualisations of oneness presents a potential tautological problem for any empirical analysis of the link between oneness and wellbeing outcomes, it is particularly important to clearly outline components of oneness which are directly related to wellbeing and those which are not directly related but which may cause wellbeing through other mechanisms.

There are few direct investigations of the relationship between self-reported oneness and well-being outcomes. While components of oneness experience may be expected to be positively correlated with well-being outcomes, these relationships need to be investigated. Having a psychometrically sound scale would help to better address these questions and further advance research focused on oneness experiences and wellbeing.

A number of models highlight potential mechanisms of oneness-related experience (e.g., Berkovich-Ohana and Glicksohn, 2014; de Castro, 2017; Dambrun et al., 2019). These models suggest that contemplative practice may diminish the usual narrative sense of self, which may in turn be associated with experiences of oneness and well-being [see Van Lente and Hogan (2020) for more details]. These theoretical accounts are also consistent with Dambrun and Ricard's (2011) model, according to which self-centeredness and an associated sense of separation from the world are linked to unhappiness, while a state of selflessness may foster feelings of connectedness and peace.

Other components of nondual or oneness experience listed in Supplementary Table S1 suggest additional mechanisms influencing wellbeing outcomes. Features of action orientation such as *non-striving* (Dambrun, 2017) and *spontaneity* (Kipper and Shemer, 2006) align with constructs in the psychology literature, including *flow* (Csikszentmihalyi, 1990), which has been linked to subjective well-being in prior empirical work (Collins et al., 2009).

Oneness may also foster a sense of *connection with everything*, which may influence social wellbeing through feelings of belonging or integration (Lamers et al., 2010). Furthermore, to the extent that oneness involves a stronger sense of *connection with others* (e.g., Leary et al., 2008), this may provide another mechanism through which oneness influences subjective and social wellbeing outcomes. At the same time, while *ego-dissolution* is often a stated goal of spiritual practices (Garfield, 2022) and can be experienced positively (Nour et al., 2016), ego-dissolution can also be a negative experience which can be associated with mental health issues (Britton et al., 2021). In the context of a comprehensive account and measurement of oneness experiences, the relationship between ego-dissolution and wellbeing requires further investigation.

Other reported aspects of oneness experience, such as *revelation of truth*, could be considered a form of eudaimonic wellbeing related to a sense of meaning in life (Lamers et al., 2010). At the same time, there is limited evidence that related *noetic* (‘direct knowing’) experiences lead to increased wellbeing (Yaden, Haidt et al., 2017; Wahbeh et al., 2022). Also, based on available research, other features of oneness experience that relate to *aliveness* (Singh and Sharma, 2018), *clarity* (Hanley and Garland, 2019), and *openness* (e.g., Sutin, 2017) may predict wellbeing. Similarly, it can be hypothesised based on available research that *equanimity and peace* (Soysa et al., 2021) would be related to wellbeing outcomes. However, a new scale of oneness experiences is needed to test these hypotheses.

### The current study

The current study follows guidelines for scale development proposed by DeVellis (2017). This involves following a series of steps in the scale development process: reviewing available literature on the constructs to be measured; generating an exhaustive scale item pool; selecting a scale item response format; evaluation of proposed scale items by content experts (who had either experienced oneness or were long-term meditators, and who had published on oneness); application of exploratory and confirmatory factor analyses to identify underlying scale factors; assessing the reliability of scales; and optimizing scale length. In addition to literature review work and analysing existing theory, DeVellis (2017) notes that qualitative work is the ideal starting point for scale construction, particularly for item development and identifying construct domains to include when generating item pools.

The conceptual framework informing scale development in the current study arose out of collective intelligence (CI) research with expert meditators who had experienced oneness (Van Lente and Hogan, 2020). A total of 41 participants, who had meditated at least 5 years over their lifetime and who had experienced oneness engaged in idea generation, clarification, and structuring across five separate CI sessions, each lasting approximately 3 h. The initial question prompting idea generation by expert meditators was: “In what ways do you see yourself as oneness, in terms of your feelings and identity, in terms of how you relate to others, and in terms of your values, goals, and actions?” The full set of ideas generated were pooled and categorised [see, e.g., Van Lente and Hogan (2020)]. Hypothesised oneness factors, based on these ideas and categories, were identified and are presented in Table 1. A total of 30 hypothesised factors emerged and are grouped across seven higher-order themes. These hypothesised factors and associated CI ideas formed the basis for item pool generation (see Supplementary Table S2 for the full set of scale items prior to factor analysis item reduction).

**Table 1:** Hypothesized oneness factors with descriptions based on qualitative research, grouped by theme.

Theme (with description)	Factor	Factor description
1. Action orientation Consists of the factors free-flow, non-striving, and spontaneity, and includes a sense of flow, non-controllingness, and comfort with spontaneity.	Free-flow	A sense that things are flowing freely
	Non-striving	A sense of no longer controlling, striving for goals, or seeking meaning
	Spontaneity	A sense that (my) actions are naturally spontaneous and do not require controlling
2. Other perception Consists of the factors connection with everything, connection with others, and being everything, and includes a focus on feeling connected to people, nature, and everything.	Connection with everything	Feeling connected to everyone and everything
	Connection with others	Feeling connected with some/ all other living beings
	Being everything	Seeing oneself as everything
3. Self-perception Comprises the factors ego-dissolution and same and different, and includes ideas that pertain to changes in the sense of self, such as recognizing self as not separate from the world, ego-dissolution, and a sense of commonality beneath differences.	Ego-dissolution	Feeling that my separate sense of self is dissolving or does not exist
	Same but different	A sense of being the same as others despite differences
4. Deconditioning and embodiment Consists of the factors aliveness, sensitivity, clarity, revelation of truth, acceptance, non-judgementality, and self-compassion, and includes ideas that pertain to feeling more alive and sensitive, being less attached to thoughts and concepts while being clearer and more insightful and being less judgmental and more accepting.	Aliveness	A sense of feeling alive alertness
	Sensitivity	A capacity to feel things precisely and intensely
	Clarity	Seeing things clearly, as they are
	Revelation of truth	A sense of having seen the essential nature of myself and reality
	Acceptance	Unconditionally accepting everything as it is
	Non-judgementality	No longer being judgemental of others
	Self-compassion	A love and acceptance of myself as a I am
5. Interpersonal emotions Comprises the factors compassion, openness, and unconditional love, and includes ideas that pertain to being open, understanding, and loving to oneself and others.	Compassion	Feeling compassion and warmth towards others
	Openness	Experiencing, feeling, and communicating in an open and unrestricted way
	Unconditional love	Loving everyone and everything as it is
6. Emotions Consists of the factors appreciation non-negativity, safety and reassurance, equanimity, and peace, and includes ideas that pertain to a sense of appreciation and joy with fewer negative emotions and a sense of unconditional peace, trust, and support.	Appreciation	Appreciating peace, people, and simple things
	Non-negativity	No longer fearing death, feeling threatened or experiencing negative emotions such as loneliness or anger or fear of rejection
	Safety and Reassurance	A feeling of being protected, cared for and supported
	Equanimity	A sense of pervasive peace regardless of what is happening
	Peace	Feeling serene, calm, and peaceful
7. Foundational orientations Comprises the factors nowness, timelessness, non-separation from everything, non-separation from others, spaciousness, part of a whole, perfect as it is, and includes ideas that pertain to spaciousness and a sense of boundaries dissolving, to timelessness, to everything being part of a whole and this whole being perfect.	Nowness	One’s attention being fully here and now
	Timelessness	A sense of both being timeless and that there is no time
	Non-separation from everything	A sense of boundaries dissolving and not being separate from anything
	Non-separation from others	A sense of boundaries dissolving and not being separate from others
	Spaciousness	A sense of limitless space and being boundless
	Part of a whole	A sense of being part of a greater whole
	Perfect as it is	Feeling that everything–- including me–- is complete as it is

In the current study, construct validity is assessed using an approach focused on convergent validity. Convergent validity considers how closely scores on the measure under development converge with scores on other measures of related constructs (Furr and Bacharach, 2008). Based on available theory and research, we hypothesise positive associations between oneness factors and measures of wellbeing, measures of mindfulness, and particular measures of personality (i.e., positive correlation with Openness and negative correlation with Neuroticism). Single-item self-reported non-dual awareness measures are also hypothesised to be correlated with oneness scale factors. The ego-dissolution factor is not expected to be as consistently correlated with these measures.

## Methods

### Design

The scale development study design involved two phases: The first phase included item generation, assessing the dimensionality of the scale using EFA, reducing the initial pool of questionnaire items, and examining the reliability and construct validity of the scale. The second phase involved confirming the factor structure derived from EFA with the same sample using a structural equation modelling (SEM) approach which also included construct validity assessment focused on evaluating hypothesized relationships between Oneness factors and wellbeing outcomes, meditation experience, mindfulness, and single item questions about oneness experience.

### Initial scale development

#### Item generation

A total of 377 items were generated on the basis of five focus group CI sessions (*N* = 41; *M_age_* = 45.37 years, *SD* = 9.79). A reduced set of between 5–10 items were selected for inclusion across 36 hypothesized components of oneness (i.e., 268 items). A number of items and categories were removed in this process, including redundant items and problematic categories. For example, *Bliss* items (*n* = 30) were removed as these have already been well represented in earlier scales and there are concerns about tautology in predicting wellbeing (Garssen et al., 2016; Koenig, 2008; MacDonald, 2017; Visser et al., 2017). On the other hand, items were added to certain categories if too few items were available. For example, items were added to the category *Being Everything* as it has previously been considered a unique aspect of oneness (e.g., Adyashanti, 2009).

#### Content validity

Scale items were subsequently reviewed by five content experts, who also rated them for their clarity and relevance. The five experts were psychology researchers who had either direct experiences of oneness [as defined in Van Lente and Hogan (2020)] or were long-term meditators, and who had all published research on elements of nonduality and oneness. The experts made a number of suggestions to increase the clarity and meaningfulness of items and suggested removal of redundant items. Overall, 132 items were rated by experts as relevant and clear and were retained for EFA. 30 constructs were selected as most important based on experts’ selections and previous literature and new items were developed closely matching existing items so that there were exactly 6 items for each of the 30 constructs (see Supplementary Table S2).

Consistent with theory and earlier research (e.g., Dambrun et al., 2019; Dorjee, 2016; Hanley et al., 2018; Schoenberg et al., 2018; Schoenberg and Vago, 2019), certain categories not thought by experts to be relevant to oneness were removed in this process, specifically, *witnessing awareness*, *body-disidentification* and *disidentification*. Also, two categories empirically thought to be related to oneness – *effective action* [see, e.g., Van Lente and Hogan (2020)] and *freedom* – were also removed because of low endorsement by experts of associated scale items. Factors with 3 or more items (27 categories) in addition to three theoretically relevant categories (*spaciousness*, *timelessness*, and *openness*) with 2 items were retained and were complemented with new items to reach 6 items. This resulted in a total of 30 proposed factors, each measured using 6 scale items.

### Further scale development

#### Procedure

Potential participants were invited to take part in an online survey of their oneness experience via university email campaigns, using the University of Galway SONA system for student research participation, and by contacting meditation, nonduality and Yoga groups in Ireland and the UK via email and Social media (Facebook, Twitter) and inviting them to participate. The study was approved by the University of Galway Research Ethics committee. All participants provided informed consent, and then completed the questionnaire packet online using the Limesurvey service.

Data collection took place between March 2019 and April 2021. Participation was anonymous and there was no remuneration for participating. There were no exclusion criteria except that participants had to be 18 years of age or over and have fluent English. As an incentive, participants could opt-in to take part in a 300 Euro draw.

#### Participants

In the survey, 762 participants completed all survey items in the online questionnaire packet. A majority had meditated at some time in their lives (65.1%) with about half (50.4%) having meditated at least a year. Descriptive statistics for the participants are presented in Table 2.

**Table 2:** Participant characteristics and descriptive statistics for main measures included in questionnaire (other than oneness).

Characteristic	Test range	Sample range	*n*	%	Mean (SD)	*α*
Sex						
Female			497	66.0		
Male			256	34.0		
Age (*n* = 762)					37.29 (17.16)	
Education (*n* = 762)						
Professional degree (e.g., MD)			28	3.7		
Doctoral degree			85	11.2		
Postgraduate degree			177	23.2		
Undergraduate degree			152	19.9		
Some undergraduate degree			124	16.3		
Post-leaving certificate course			46	6.0		
Leaving certificate (or equivalent)			130	17.1		
Junior certificate or less (or equivalent)			8	1.1		
Would rather not say			12	1.6		
Employment						
Employed			274	36.0		
Self-employed			106	13.9		
Student			268	35.1		
Homemaker			14	1.8		
Retired			52	6.8		
Unemployed			31	4.1		
Unable to work			7	0.9		
Would rather not say			10	1.3		
FFMQ mindfulness						
Observe (8)	8–40	8–40			3.80 (0.70)	0.84
Describe (8)	8–40	9–40			3.57 (0.82)	0.90
Act with awareness (8)	8–40	8–40			3.14 (0.82)	0.90
Non-judgement (8)	8–40	8–40			3.42 (0.97)	0.93
Nonreact (7)	7–35	7–35			3.27 (0.80)	0.88
MHC-SF wellbeing						
Hedonic						
Emotional wellbeing (3)	3–18	3–18			4.65 (1.02)	0.87
Eudaimonic						
Social wellbeing (5)	5–30	5–30			3.72 (1.20)	0.82
Psychological wellbeing (6)	6–36	8–36			4.47 (1.09)	0.87
BFI-10 personality						
Extraversion (2)	1–10	1–10			3.25 (1.04)	0.61 (*r* = 0.44***)
Agreeableness (2)	1–10	1–10			3.67 (0.92)	0.43 (*r* = 0.28***)
Conscientiousness (2)	1–10	1–10			3.62 (0.96)	0.49 (*r* = 0.34***)
Neuroticism (2)	1–10	1–10			2.89 (1.16)	0.66 (*r* = 0.50***)
Openness (2)	1–10	1–10			3.83 (0.93)	0.27 (*r* = 0.17***)
Any meditation experience						
Never			266	34.9		
Less than a year			112	14.7		
More than a year			384	50.4		
Meditation quantity						
1 Total lifetime hours					6854.45 (17284.27)	
2 Number of lifetime incidences					2718.05 (4026.57)	
3 Total number of months of practice					130.23 (150.06)	
4 Number of practice sessions per week					3.91 (2.14)	
5 Average length of session (minutes)					28.32 (25.87)	

FFMQ, five facet mindfulness questionnaire; MHC-SF, mental health continuum-SF; BFI-10, big five inventory-10 personality inventory; M, mean; SD, standard deviation; *α*, Cronbach’s alpha coefficient; r, Pearson’s r correlation (alterative to Cronbach’s alpha for two item scales).

#### Materials

In addition to demographic questions (i.e., age, gender, educational, and occupational status), participants completed the following measures:

##### Mindfulness

Mindfulness was assessed using the 39 item Five Facet Mindfulness Questionnaire (FFMQ; Baer et al., 2006). All FFMQ items are scored on a 5-point scale (1 = *never or very rarely true*, 5 = *very often or always true*). *Observe*, *Describe*, *Actaware*, and *Nonjudge* facets are each measured by 8 items, whereas *Nonreact* is measured with 7 items. *Observe* items primarily relate to paying attention to sensations and perceptions, but also noticing their effects on emotions, thought and behaviour. *Describe* relates to being able to put one’s sensations, feelings, and thoughts into words. *Actaware* involves being able to pay attention to and stay focused on what one is doing. *Nonjudge* involves not judging oneself for having ‘wrong’ (i.e., irrational or bad) thoughts and feelings and not thinking one should not have them. *Nonreact* relates to being able to experience distressing feelings, thoughts and images without being taken over by or reacting to them. The Cronbach’s *α*s of the five scales in the current study were 0.84 for Observe, 0.90 for Describe, 0.93 for Actaware, 0.93 for Nonjudge, and 0.88 for Nonreact.

##### Wellbeing

Wellbeing was assessed using the 14 item Mental Health Continuum (Short Form; MHC-SF) wellbeing scale (Lamers et al., 2010). This scale measures wellbeing across three levels, including (a) feelings of happiness (emotional/subjective wellbeing), (b) “positive social functioning” (social wellbeing), a construct which measures the sense that one belongs to and can make a positive contribution to what one perceives to be a coherent, good, and improving society, and (c) “positive psychological functioning” (psychological wellbeing), a construct that includes measures of self-acceptance, personal growth, purpose in life, environmental mastery, autonomy, and positive relations with others (Ryff and Keyes, 1995).

Items of the MHC scale are scored on a 5-point frequency scale (1 = *never*, 5 = *every day*) and the Cronbach’s α reliability for this overall scale was 0.92. For the wellbeing subscales Cronbach’s α reliabilities were: *Emotional* = 0.87; *Psychological* = 0.87; *Social* = 0.82.

##### Personality

Personality was measured using the 10-item short version of the Big-Five Inventory (Rammstedt and John, 2007). All items are scored on a 5-point scale (1 = *Disagree strongly*, 5 = *Agree strongly*). The Cronbach’s α reliability ranged from low for openness (0.27) to somewhat higher for extraversion (0.61).

##### Meditation-related questions

A number of questions captured respondents’ meditation experience. These questions included:

- *Time spent meditating*, assessed using the following questions: (1) How long have you engaged in meditation/contemplative practices (including ‘moving meditation’ such as yoga)? Possible answers were: (a) *Never*, (b) *less than a year*; (c) *more than a year*.
- A follow up question asked for details of the *number of months and years of meditation* (for those who meditated more than a year) and months of meditation (for those who meditated less than a year).
- Average number of meditation sessions per week and average length of each session

Based on these variables the following were calculated: (1) Total lifetime meditation hours, (2) Number of lifetime incidences, (3) Total number of months of practice.

##### Self-reported nondual awareness frequency and intensity

These variables were measured using three questions asked using two definitions of nondual awareness. The definitions were: “A sense of unified experience where you feel a sense of oneness with the world and other people and you don’t feel separate.”, “A sense of unified experience in which the usual subject/object distinctions are no longer dominant.” The first question which addressed frequency using a 5-point Likert scale asked: “Based on this [first/second] definition, how often do you experience nondual awareness? The second and third questions addressed intensity, also using a 5-point Likert scale which asked: “Based on this [first/second] definition, to what extent do you agree or disagree with the following statements:” which were: “(a) I have a deep experience of nondual awareness in my everyday life”, and “(b) I have a profound recognition of nondual awareness in my everyday life.

##### Oneness

The oneness measure which is the focus for scale development in the current study was based on collective intelligence work (Van Lente and Hogan, 2020). 179 expert-reviewed oneness items were included (see Supplementary Table S2). All items are scored on a 5-point Likert scale (1 = *never or very rarely true*, 5 = *very often or always true*). Descriptive statistics and Cronbach’s alpha for all measures included in this study are presented in Table 2.

## Results

### Exploratory factor analysis

An Exploratory Factor Analysis (Principal Axis Factoring) was carried out using SPSS version 26. Promax Rotation with Kaiser Normalisation was employed. The Kaiser Meyer-Olkin measure (KMO = 0.96), and a significant Bartlett’s test of sphericity [X^2^(3486) = 51568.24, *p* < 0.001] indicated suitability of the dataset for factor analysis. Factor retention decisions were made on the basis of Horn’s parallel analysis (Horn, 1965), as well as conceptual considerations (e.g., having at least 3 items per factor). Consistent with Worthington and Whittaker (2006) guidelines, items were retained if they had loadings in excess of 0.50^1^, no cross-loadings above 0.32, and item communalities over 0.40. Each factor was assessed for the presence of redundant items, and within factor inter-item correlations were between 0.30 and 0.90. Item-total correlations were required to be above 0.30 to allow averaging of factor scores without applying item weights (Field, 2009).

#### Reliability

Scale reliability was assessed using Cronbach, (1951), with values of at least 0.70 indicating acceptable internal consistency (Nunnally, 1978).

According to Worthington and Whittaker (2006), the related but distinct processes of factor analysis and item deletion should be undertaken iteratively. In the current case, this process involved undertaking an EFA, and removing items from the analysis (based on the criteria above), until it was no longer necessary to remove items based on Worthington and Whittaker (2006) criteria or based on parallel analysis criteria (Costello and Osborne, 2005; Shonrock-Adema et al., 2009). Finally, in order to create a scale with a manageable number of items, factors with more than 4 items were further reduced to include only the top four highest loading items. This process resulted in the retention of 84 items remaining across 21 factors. Factor analysis of these 84 items was checked against Worthington and Whittaker (2006) criteria, which suggested no deletions. Finally, a parallel analysis (O’Connor, 2000) using permutations of the raw dataset was applied to the final dataset. The number of factors based on parallel analysis (*n* = 23) exceeded the number generated by EFA, suggesting that no remaining factors needed to be removed. Results of the EFA are shown in Table 3. Supplementary Table S2 groups all factors and items by reference to higher-order factors which are the theoretical themes described in Table 1. Supplementary Table S3 presents the 84 scale items retained in the EFA, grouped by factor, together with instructions for administration.

**Table 3:** Results of EFA and psychometric properties of the 21 factors of the Oneness Scale.

	%	Loading	Loading	*α*	Eigen	*M* (SD)
Factor	Variance	(Range)	(*M*)		Value	
1. Part of a whole and connection with everything (8) [POAW_CWE]	34.13	0.72–0.88	0.80	0.94	28.67	3.54(1.07)
2. Ego dissolution (4)	6.36	0.82–0.96	0.90	0.95	5.34	2.56(1.26)
3. Clarity (4)	4.03	0.76–0.94	0.85	0.92	3.39	3.57(0.99)
4. Nowness (4)	3.26	0.74–0.91	0.84	0.92	2.74	3.8(1.01)
5. Safety and reassurance (4)	2.79	0.75–0.85	0.78	0.88	2.34	3.94(0.93)
6. Non-judgementality (4)	2.56	0.68–0.93	0.82	0.89	2.15	3.64(1.00)
7. Timelessness (4)	2.33	0.71–0.86	0.82	0.90	1.96	2.82(1.24)
8. Spontaneity (4)	2.16	0.57–0.86	0.72	0.84	1.82	3.50(0.97)
9. Peace (4)	1.97	0.87–0.90	0.88	0.94	1.66	3.48(1.11)
10. Compassion (4)	1.91	0.59–0.85	0.75	0.84	1.61	4.39(0.67)
11. Self-compassion (4)	1.77	0.74–0.87	0.80	0.90	1.48	3.73(1.07)
12. Non-striving (4)	1.71	0.55–0.89	0.72	0.82	1.44	2.73(1.12)
13. Spaciousness (4)	1.59	0.81–0.88	0.84	0.92	1.34	3.06(1.22)
14. Acceptance (4)	1.56	0.77–0.88	0.81	0.90	1.31	3.44(1.11)
15. Non-separation from others (4)	1.51	0.71–0.94	0.80	0.87	1.27	3.19(1.10)
16. Revelation of truth (4)	1.48	0.60–0.88	0.76	0.88	1.24	3.33(1.10)
17. Flow (4)	1.37	0.72–0.84	0.78	0.88	1.15	3.60(1.00)
18. Perfect as it is (3)	1.30	0.80–0.89	0.83	0.92	1.09	2.46(1.30)
19. Openness (3)	1.26	0.71–0.83	0.76	0.81	1.05	4.11(0.89)
20. Appreciation (3)	1.23	0.63–0.78	0.71	0.78	1.04	4.52(0.65)
21. Same and different (3)	1.21	0.54–0.80	0.71	0.82	1.02	3.98(0.91)
Oneness—total scale (84 items)				0.98		3.48(0.69)

Sample size (*N*) = 762; *α*, Cronbach’s alpha coefficient; *M*, mean; SD, standard deviation.

### Confirmatory factor analysis

Using the same sample, a CFA was conducted using Structural Equation Modeling (SEM) in Amos version 25 (Arbuckle, 2020). Model fit was assessed using a number of indices. Firstly, a non-significant chi square test is indicative of a well-fitting model. The normed chi-square (Q) was also assessed (i.e., the chi square index divided by the degrees of freedom), with acceptable criteria ranging from less than 2 (Ullman, 2001) to less than 5 (Schumacker and Lomax, 2004). The comparative fit index (CFI) was also evaluated: values of 0.90 and 0.95 reflect a range from acceptable to excellent fit to the data (Kenny and McCoach, 2003). Finally, the root mean square error of approximation (RMSEA) was used, with values between 0.05 and 0.09 indicating adequate model fit and values below 0.05 indicating a very good fit (Hu and Bentler, 1999). Modification indices available in Amos CFA can be used to identify misspecification in the model. Decisions regarding modifications were based on theoretical and psychometric considerations of item and scale content. Error residuals were not permitted to covary, but items were eliminated if they had low factor loadings (i.e., standardized regression coefficients <0.60), or if modification indices suggested they had significant loadings (>0.30) with unintended latent factors (Byrne, 2010).

The 21-factor solution identified using EFA was tested, with the 84 scale items loading onto their respective factors in a CFA structure in AMOS. This initial measurement model provided a very good fit: χ^2^(3192) = 6423.29, *p* < 0.001, Q = 2.01, CFI = 0.94, RMSEA = 0.036 (90% CI, 0.035–0.038). All items loaded above 0.60 (the lowest being one loading on the *compassion* factor of 0.62). The 84 items of the Oneness scale and their beta weights (*β*), as well as the proportion of variance in the latent construct explained by that item (*r*^2^) are reported in Table 4.

**Table 4:** The oneness scale, internal consistency of 21 subscales and psychometric properties of 84 final scales items.

Oneness scale factor	Oneness scale item	Beta	r^2^	Cronbach’s Alpha	M (SD)
1 ConnectionWithEvery_1	Feeling a sense of connection to everyone and everything	0.77	0.59	0.94	3.54 (1.07)
1 ConnectionWithEvery_3	A feeling of connection to everything	0.89	0.78	0.94	3.54 (1.07)
1 ConnectionWithEvery_4	Experiencing connection with all that is	0.82	0.67	0.94	3.54 (1.07)
1 ConnectionWithEvery_5	Feeling connected with all of nature	0.73	0.53	0.94	3.54 (1.07)
1 PartOfAWhole_1	A sense of being an intrinsic part of all that is	0.8	0.64	0.94	3.54 (1.07)
1 PartOfAWhole_2	A sense that I am part of an all-pervading consciousness	0.72	0.51	0.94	3.54 (1.07)
1 PartOfAWhole_5	Feeling that my individual life is part of a greater whole	0.81	0.66	0.94	3.54 (1.07)
1 PartOfAWhole_6	Feeling that I am part of a greater unity or oneness	0.87	0.75	0.94	3.54 (1.07)
2 Egodissolution_2	A feeling that my self is dissolving	0.96	0.92	0.95	2.56 (1.26)
2 Egodissolution_3	A sense of my separate identity being dissolved away	0.82	0.67	0.95	2.56 (1.26)
2 Egodissolution_4	A sense of me dissolving	0.91	0.82	0.95	2.56 (1.26)
2 Egodissolution_5	A sense of dissolution of the self	0.91	0.82	0.95	2.56 (1.26)
3 Clarity_1	A feeling of clarity	0.94	0.87	0.92	3.57 (0.99)
3 Clarity_3	Seeing things clearly	0.78	0.6	0.92	3.57 (0.99)
3 Clarity_4	A sense of clarity	0.93	0.86	0.92	3.57 (0.99)
3 Clarity_5	Everything is perceived with clarity	0.76	0.58	0.92	3.57 (0.99)
4 Nowness_2	Being in the moment	0.85	0.72	0.92	3.8 (1.01)
4 Nowness_3	A sense of being focused on the present	0.74	0.55	0.92	3.8 (1.01)
4 Nowness_5	A sense of being present in this moment	0.85	0.72	0.92	3.8 (1.01)
4 Nowness_6	Being here in the present	0.91	0.83	0.92	3.8 (1.01)
5 Safetyandreassurance_1	A feeling of support and love	0.75	0.56	0.88	3.94 (0.93)
5 Safetyandreassurance_2	Feeling safe	0.76	0.58	0.88	3.94 (0.93)
5 Safetyandreassurance_4	A sense of being taken care of	0.85	0.72	0.88	3.94 (0.93)
5 Safetyandreassurance_5	A sense of safety and reassurance	0.77	0.59	0.88	3.94 (0.93)
6 Non-judgementality_3	No longer being judgemental of others	0.93	0.86	0.89	3.64 (1)
6 Non-judgementality_4	Feeling less judgemental to others	0.68	0.46	0.89	3.64 (1)
6 Non-judgementality_6	No longer judging others behaviour	0.87	0.75	0.89	3.64 (1)
7 Timelessness_1	A sense of timelessness	0.85	0.72	0.9	2.82 (1.24)
7 Timelessness_2	A sense of being timeless	0.86	0.74	0.9	2.82 (1.24)
7 Timelessness_4	A sense of there being no time	0.71	0.51	0.9	2.82 (1.24)
7 Timelessness_5	A sense of being outside of time	0.84	0.71	0.9	2.82 (1.24)
8 Spontaneity_2	My spontaneous actions feel natural	0.61	0.37	0.84	2.82 (1.24)
8 Spontaneity_3	A sense of my actions happening spontaneously	0.86	0.74	0.84	2.82 (1.24)
8 Spontaneity_4	A sense that my spontaneous actions do not need to be controlled	0.58	0.33	0.84	2.82 (1.24)
8 Spontaneity_5	A sense of spontaneity in my actions	0.85	0.72	0.84	2.82 (1.24)
9 Peace_1	Feeling tranquil	0.9	0.81	0.94	3.48 (1.11)
9 Peace_2	Feeling serene	0.87	0.76	0.94	3.48 (1.11)
9 Peace_5	A strong sense of peace and calm	0.87	0.76	0.94	3.48 (1.11)
9 Peace_6	Feelings of peace and calm	0.88	0.77	0.94	3.48 (1.11)
10 Compassion_1	Feelings of empathy and compassion	0.82	0.68	0.84	4.39 (0.67)
10 Compassion_2	A feeling of great compassion	0.85	0.73	0.84	4.39 (0.67)
10 Compassion_3	Feeling great warmth towards others	0.73	0.53	0.84	4.39 (0.67)
10 Compassion_4	A happiness for others being happy	0.59	0.35	0.84	4.39 (0.67)
11 Selfcompassion_1	A feeling of self-compassion	0.74	0.55	0.9	3.73 (1.07)
11 Selfcompassion_2	A deep acceptance and love for what I am	0.81	0.65	0.9	3.73 (1.07)
11 Selfcompassion_5	Acceptance of my personality	0.78	0.61	0.9	3.73 (1.07)
11 Selfcompassion_6	Loving myself the way I am	0.87	0.76	0.9	3.73 (1.07)
12 Nonstriving_2	No longer striving	0.89	0.78	0.82	2.73 (1.12)
12 Nonstriving_3	Not forcing things to go my way	0.55	0.3	0.82	2.73 (1.12)
12 Nonstriving_5	No longer searching for meaning	0.62	0.39	0.82	2.73 (1.12)
12 Nonstriving_6	A sense of no longer having goals	0.81	0.66	0.82	2.73 (1.12)
13 Spaciousness_1	A sense of being boundless	0.81	0.66	0.92	3.06 (1.22)
13 Spaciousness_2	A sense of open, infinite space	0.85	0.71	0.92	3.06 (1.22)
13 Spaciousness_5	Experiences of boundarylessness	0.81	0.65	0.92	3.06 (1.22)
13 Spaciousness_6	A sense of limitless space	0.88	0.77	0.92	3.06 (1.22)
14 Acceptance_1	A deep sense of acceptance	0.77	0.59	0.9	3.44 (1.11)
14 Acceptance_2	A deep sense of acceptance no matter how good or how difficult the situation	0.81	0.66	0.9	3.44 (1.11)
14 Acceptance_4	Accepting everything as it is	0.88	0.77	0.9	3.44 (1.11)
14 Acceptance_5	Allowing everything to be as it is	0.79	0.63	0.9	3.44 (1.11)
15 NonSeparationOthers_1	A disappearance of boundaries between self and other	0.71	0.5	0.89	3.19 (1.1)
15 NonSeparationOthers_3	A sense of not being separate from others	0.94	0.88	0.89	3.19 (1.1)
15 NonSeparationOthers_5	No longer feeling separate from others	0.83	0.69	0.89	3.19 (1.1)
15 NonSeparationOthers_6	Knowing that I am ultimately not separate from others	0.72	0.52	0.89	3.19 (1.1)
16 RevelationOfTruth_1	Realising ultimate truths	0.88	0.78	0.88	3.33 (1.1)
16 RevelationOfTruth_2	Realising who I am at the deepest level	0.6	0.35	0.88	3.33 (1.1)
16 RevelationOfTruth_5	Seeing the essential nature of reality	0.77	0.59	0.88	3.33 (1.1)
16 RevelationOfTruth_6	A sense that the core nature of things has been revealed	0.78	0.61	0.88	3.33 (1.1)
17 Flow_1	A feeling of flow	0.84	0.71	0.89	3.6 (1)
17 Flow_2	A feeling of life flowing freely	0.81	0.65	0.89	3.6 (1)
17 Flow_3	A feeling of life flowing spontaneously	0.74	0.55	0.89	3.6 (1)
17 Flow_5	A sense of flow even in the face of resistance	0.72	0.52	0.89	3.6 (1)
18 PerfectAsItIs_1	A recognition that everything is perfect	0.89	0.78	0.92	2.46 (1.3)
18 PerfectAsItIs_2	A sense that everything is already perfect and complete just as it is	0.8	0.64	0.92	2.46 (1.3)
18 PerfectAsItIs_5	Feeling that the world is perfect as it is	0.82	0.67	0.92	2.46 (1.3)
19 Openness_2	Communicating openly	0.83	0.69	0.81	4.11 (0.89)
19 Openness_4	Being open to feel my feelings	0.71	0.5	0.81	4.11 (0.89)
19 Openness_5	Feelings of openness	0.74	0.54	0.81	4.11 (0.89)
20 Appreciation_1	A feeling of appreciating simple things	0.63	0.4	0.78	4.52 (0.65)
20 Appreciation_4	Appreciating contentment and peace	0.78	0.61	0.78	4.52 (0.65)
20 Appreciation_6	Savouring moments of quiet and silence	0.73	0.54	0.78	4.52 (0.65)
21 SameAndDifferent_2	Feeling one with others despite differences	0.54	0.3	0.82	3.98 (0.91)
21 SameAndDifferent_4	A sense of being the same despite our differences	0.8	0.64	0.82	3.98 (0.91)
21 SameAndDifferent_5	A sense of commonality underneath our differences	0.79	0.63	0.82	3.98 (0.91)

Intercorrelations between the 21 subscales (see Table 5) suggest they measure related, yet distinct, constructs (*Mean* = 0.40; *SD* = 0.11; Range = 0.07–0.65). Inspection of the scale items led to the following factor interpretations. Factor 1, *Part of a Whole & Connection with Everything* measures a sense that everything – including oneself – is an intrinsic part of a whole and is connected to everything. Factor 2, *Ego dissolution* focuses on a sense that one’s (separate) self is dissolving or has dissolved. Factor 3, *Clarity* captures the sense that one can see and feel things clearly without distortion. Factor 4, *Nowness* captures a sense of being here now. Factor 5, *Safety and Reassurance* taps into a sense of unconditional safety, nurturance, and support. Factor 6, *Non-judgementality* reflects no longer being judgemental of others. Factor 7, *Timelessness* reflects the sense that the conventional three-part division of time into past, present, and future no longer fits with one’s experience. Factor 8, *Spontaneity* measures a sense of naturalness in one’s actions, where control feels unnecessary. Factor 9, *Peace* captures an internal sense of peace and calm. Factor 10, *Compassion* refers to being there for others when they are suffering and feeling joy in others existence and wellbeing. Factor 11, *Self-compassion* captures a sense of love and acceptance of myself as I am. Factor 12, *Non-striving* captures a sense of allowing things to be as they are. Factor 13, *Spaciousness* reflects a sense of boundaries dissolving or having dissolved, to reveal unbounded spaciousness. Factor 14, *Acceptance* reflects a sense of unconditionally accepting everything as it is. Factor 15, *Non- Separation from Others* taps into a sense of boundaries dissolving or having dissolved, to reveal that others are not separate. Factor 16, *Revelation of Truth* measures the profound sense of awareness and insight in relation to fundamental reality. Factor 17, *Flow* taps into a sense of action being free, spontaneous, and unrestrainable. Factor 18, *Perfect as it is* reflects a sense that everything – including oneself – is perfect as it is. Factor 19, *Openness* measures feeling open, communicating openly and being open to one’s feelings. Factor 20, *Appreciation* measures appreciating wellbeing, peace, silence, and simple things. Factor 21, *Same and different* indicates feeling sameness with others despite differences.

**Table 5:** Intercorrelations of 21 oneness factors remaining after exploratory factor analysis.

Factor	POAW/CWE	Ego-dissolution	Clarity	Nowness	Safety and reassurance	Non-judgementality	Timelessness	Spontaneity	Peace	Compassion	Self-compassion	Non-striving	Spaciousness	Acceptance	Non-separation from others	Revelation of truth	Flow	Perfect as it is	Openness	Appreciation
Ego-dissolution	0.39	1																		
Clarity	0.42	0.17	1																	
Nowness	0.48	0.24	0.53	1																
Safety and reassurance	0.31	.07^a^	0.38	0.39	1															
Non-judge-mentality	0.41	0.28	0.34	0.31	0.26	1														
Timelessness	0.49	0.53	0.26	0.31	0.13	0.3	1													
Spontaneity	0.44	0.27	0.37	0.45	0.31	0.33	0.33	1												
Peace	0.56	0.29	0.56	0.60	0.46	0.36	0.39	0.45	1											
Compassion	0.42	0.15	0.27	0.32	0.39	0.37	0.21	0.29	0.33	1										
Self-compassion	0.54	0.15	0.52	0.55	0.45	0.38	0.31	0.41	0.57	0.34	1									
Non-striving	0.35	0.45	0.31	0.29	0.13	0.32	0.40	0.32	0.38	0.09	0.27	1								
Spaciousness	0.65	0.45	0.44	0.49	0.33	0.35	0.55	0.43	0.55	0.31	0.48	0.42	1							
Acceptance	0.57	0.32	0.50	0.55	0.38	0.47	0.38	0.47	0.59	0.38	0.59	0.40	0.50	1						
Non-separation from others	0.63	0.41	0.37	0.42	0.37	0.42	0.48	0.41	0.47	0.35	0.45	0.39	0.55	0.49	1					
Revelation of truth	0.63	0.34	0.52	0.49	0.28	0.37	0.43	0.38	0.51	0.31	0.49	0.32	0.55	0.50	0.49	1				
Flow	0.57	0.31	0.49	0.57	0.40	0.31	0.40	0.55	0.58	0.34	0.55	0.35	0.53	0.55	0.46	0.54	1			
Perfect as it is	0.53	0.43	0.40	0.43	0.30	0.41	0.48	0.45	0.53	0.22	0.49	0.47	0.52	0.58	0.49	0.48	0.50	1		
Openness	0.46	0.12	0.38	0.45	0.34	0.31	0.25	0.38	0.42	0.44	0.49	0.18	0.36	0.42	0.37	0.39	0.44	0.34	1	
Appreciation	0.44	0.13	0.31	0.40	0.28	0.25	0.19	0.31	0.41	0.44	0.43	0.19	0.31	0.4	0.29	0.30	0.41	0.25	0.42	1
Same and different	0.56	0.25	0.28	0.34	0.31	0.39	0.36	0.33	0.37	0.40	0.42	0.27	0.43	0.43	0.50	0.40	0.43	0.39	0.37	0.35

POAW/CWE, part of a whole/connection with everything.

^a^ Correlation is not-significant, all other correlations significant at the 0.01 level (2-tailed).

### Higher-order factor structure

To examine whether the 21 first-order factors could be represented by a smaller number of broader dimensions, a higher-order confirmatory factor analysis was conducted. The 21 first-order factors were specified as indicators of six higher-order factors: Foundations (POAW, Nowness, Timelessness, Non-Separation from Others, Spaciousness, Perfect as It Is), Self (Ego Dissolution, Same and Different), Deconditioning (Clarity, Revelation of Truth, Acceptance, Nonjudgement, Self-Compassion), Emotions (Safety and Reassurance, Peace, Appreciation), Action (Spontaneity, Non-Striving, Flow), and Interpersonal (Compassion, Openness). These six higher-order factors correspond to the overarching thematic structure identified in prior qualitative research (Van Lente and Hogan, 2020), providing theoretical grounding for this structure.

The higher-order model demonstrated acceptable fit: χ^2^(3,355) = 6607.60, *p* < 0.001, χ^2^/df = 1.97, CFI = 0.935, RMSEA = 0.036 (90% CI: 0.034–0.037). Standardised factor loadings of the 21 first-order factors on the six higher-order factors are presented in Table 6. All loadings were statistically significant (*p* < 0.001) and ranged from 0.487 (Ego Dissolution on Self) to 0.826 (Acceptance on Deconditioning), with the majority exceeding 0.60. These results suggest that the higher-order structure may provide a coherent and interpretable summary of the 21 first-order dimensions, and offers a more parsimonious framework for researchers who seek to measure oneness experience across domain levels. As with the first-order CFA, these results should be considered preliminary pending replication with an independent sample.

**Table 6:** Standardised factor loadings of first-order factors on higher-order factors in the six-factor higher-order model.

Higher-order factor	First-order factor	Loading
Foundations	POAW/CWE	0.813
	Nowness	0.677
	Timelessness	0.644
	Non-separation from others	0.757
	Spaciousness	0.798
	Perfect as it is	0.736
Self	Ego dissolution	0.487
	Same and different	0.569
Deconditioning	Clarity	0.679
	Revelation of truth	0.743
	Acceptance	0.826
	Nonjudgement	0.559
	Self-compassion	0.780
Emotions	Safety and reassurance	0.603
	Peace	0.819
	Appreciation	0.625
Action	Spontaneity	0.667
	Non-striving	0.621
	Flow	0.825
Interpersonal	Compassion	0.652
	Openness	0.780

All loadings significant at *p* < 0.001. POAW/CWE, part of a whole/connection with everything.

### Reliability

*Internal Consistency*: Results support the reliability of the Oneness scale. Cronbach’s alpha (Cronbach, (1951)) coefficients for all subscales were above the level of acceptable internal consistency (Nunnally, 1978) and consistently higher (0.78–0.95) (See Table 4). The reliability of the overall scale is 0.98.

### Convergent validity

To evaluate the convergent validity of the constructs being measured, proposed relationships between subscales of the oneness scale and wellbeing outcomes, mindfulness predictors, personality, and single-item nondual awareness measures, were assessed using Pearson’s correlations.

#### Wellbeing

Hypothesised positive associations were confirmed, and the results are presented in Table 7. Psychological wellbeing was, on average, most strongly associated with the oneness factors (*M* = 0.375; range = 0.056–0.646), while social wellbeing (*M* = 0.327; range = 0.026–0.484) was the least strongly associated. All factors, except *ego-dissolution*, were significantly positively associated with the three types of wellbeing. *Self-compassion* had the highest average correlation (*M* = 0.574) across the three wellbeing types, and *ego-dissolution* had the lowest average correlation (*M* = 0.036). About three-quarters (76%) of the correlations had at least medium-sized effects (*r* > = 0.30; Cohen, 1992).

**Table 7:** Bivariate Pearson correlations between 21 oneness scale factors and wellbeing measures.

Factor	Theme name	Theme number	Emotional wellbeing	Social wellbeing	Psychological wellbeing
POAW/CWE	Foundational orientations / other-perception	7	0.446^**^	0.422^**^	0.480^**^
Ego-dissolution	Self-perception	3	0.027	0.026	0.056
Clarity	Deconditioning and embodiment	4	0.462^**^	0.385^**^	0.482^**^
Nowness	Foundational orientations	7	0.434^**^	0.369^**^	0.429^**^
Safety and reassurance	Emotions	6	0.454^**^	0.390^**^	0.425^**^
Non-judgementality	Deconditioning and embodiment	4	0.262^**^	0.293^**^	0.279^**^
Timelessness	Foundational orientations	7	0.199^**^	0.202^**^	0.228^**^
Spontaneity	Action orientation	1	0.336^**^	0.270^**^	0.300^**^
Peace	Emotions	6	0.529^**^	0.430^**^	0.458^**^
Compassion	Interpersonal emotions	5	0.296^**^	0.309^**^	0.315^**^
Self-compassion	Deconditioning and embodiment	4	0.593^**^	0.484^**^	0.646^**^
Non-striving	Action orientation	1	0.206^**^	0.118^**^	0.136^**^
Spaciousness	Foundational orientations	7	0.353^**^	0.323^**^	0.361^**^
Acceptance	Deconditioning and embodiment	4	0.453^**^	0.344^**^	0.409^**^
Non-separation from others	Foundational orientations	7	0.388^**^	0.367^**^	0.401^**^
Revelation of truth	Deconditioning and embodiment	4	0.364^**^	0.374^**^	0.440^**^
Flow	Action orientation	1	0.453^**^	0.363^**^	0.459^**^
Perfect as it is	Foundational orientations	7	0.386^**^	0.375^**^	0.368^**^
Openness	Interpersonal emotions	5	0.426^**^	0.366^**^	0.496^**^
Appreciation	Emotions	6	0.321^**^	0.286^**^	0.347^**^
Same and different	Self-perception	3	0.364^**^	0.379^**^	0.364^**^

**p < 0.01. Correlation is significant at the 0.01 level (2-tailed).

#### Mindfulness

Another way of evaluating convergent validity is by examining proposed relationships with mindfulness. These results are presented in Table 8. In terms of Facets, *non-reactiveness* had the highest average correlation (*M* = 0.368), while *describe* had the lowest (*M* = 0.202). Approximately 81% of oneness components demonstrated at least one moderate correlation (*r* ≥ 0.30) with a mindfulness facet, while 48% demonstrated a moderate correlation on average across all five mindfulness facets. Among the oneness components, *Self-compassion* had the highest average correlation (*M* = 0.410), while *ego-dissolution* had the lowest average correlation with mindfulness facets (*M* = 0.144).

**Table 8:** Bivariate Pearson correlations between 21 oneness scale factors and FFMQ mindfulness measures.

Factor	Theme	Observe	Describe	Act with awareness	Non-judgement	Non-react
POAW/CWE	7&2	0.459^**^	0.297^**^	0.356^**^	0.347^**^	0.451^**^
Ego-dissolution	3	0.208^**^	0.014	0.148^**^	0.085^*^	0.267^**^
Clarity	4	0.265^**^	0.283^**^	0.367^**^	0.383^**^	0.424^**^
Nowness	7	0.332^**^	0.230^**^	0.393^**^	0.343^**^	0.427^**^
Safety and reassurance	7	0.144^**^	0.135^**^	0.164^**^	0.244^**^	0.221^**^
Non-judgementality	4	0.212^**^	0.131^**^	0.222^**^	0.248^**^	0.270^**^
Timelessness	7	0.249^**^	0.115^**^	0.187^**^	0.224^**^	0.325^**^
Spontaneity	1	0.278^**^	0.154^**^	0.241^**^	0.326^**^	0.335^**^
Peace	6	0.332^**^	0.213^**^	0.399^**^	0.442^**^	0.521^**^
Compassion	5	0.298^**^	0.189^**^	0.132^**^	0.097^**^	0.142^**^
Self-compassion	4	0.321^**^	0.294^**^	0.428^**^	0.524^**^	0.483^**^
Non-striving	1	0.171^**^	0.117^**^	0.259^**^	0.301^**^	0.368^**^
Spaciousness	7	0.367^**^	0.180^**^	0.321^**^	0.278^**^	0.450^**^
Acceptance	4	0.273^**^	0.152^**^	0.301^**^	0.331^**^	0.445^**^
Non-separation others	7	0.287^**^	0.186^**^	0.304^**^	0.277^**^	0.408^**^
Revelation of truth	4	0.333^**^	0.258^**^	0.306^**^	0.273^**^	0.379^**^
Flow	1	0.360^**^	0.269^**^	0.305^**^	0.328^**^	0.430^**^
Perfect as it is	7	0.227^**^	0.148^**^	0.326^**^	0.383^**^	0.413^**^
Openness	5	0.367^**^	0.430^**^	0.318^**^	0.336^**^	0.342^**^
Appreciation	6	0.399^**^	0.232^**^	0.226^**^	0.221^**^	0.299^**^
Same and different	3	0.312^**^	0.209^**^	0.288^**^	0.253^**^	0.324^**^

*p < 0.05 (2-tailed).

**p < 0.01 (2-tailed).

#### Personality

Table 9 shows correlations of oneness factors with personality variables. Average correlations were strongest in the positive direction with agreeableness (*M* = 0.272) and in the negative direction with neuroticism (*M* = −0.300). *Openness* (*M* = 0.064) showed the fewest significant correlations, being significantly correlated with approximately half (48%) of the oneness factors. Self-compassion was most strongly associated with personality variables (mean |r| = 0.261), while *ego-dissolution* was most weakly associated (mean |r| = 0.078).

**Table 9:** Bivariate spearman correlations between 21 oneness scale factors and personality variables.

Factor	Extraversion	Agreeable-ness	Conscientious-ness	Neuroticism	Openness
POAW/CWE	0.148^**^	0.320^**^	0.187^**^	−0.331^**^	0.128^**^
Ego-dissolution	−0.011	0.128^**^	0.045	−0.180^**^	0.049
Clarity	0.120^**^	0.224^**^	0.213^**^	−0.385^**^	0.034
Nowness	0.095^**^	0.286^**^	0.184^**^	−0.412^**^	0.044
Safety and reassurance	0.209^**^	0.323^**^	0.107^**^	−0.226^**^	0.015
Non-judgementality	0.119^**^	0.374^**^	0.092^*^	−0.213^**^	0.077^*^
Timelessness	0.055	0.156^**^	0.084^*^	−0.187^**^	0.074^*^
Spontaneity	0.168^**^	0.309^**^	0.065	−0.274^**^	0.055
Peace	0.079^*^	0.316^**^	0.211^**^	−0.465^**^	0.059
Compassion	0.220^**^	0.314^**^	0.141^**^	−0.109^**^	0.102^**^
Self-compassion	0.213^**^	0.310^**^	0.247^**^	−0.464^**^	0.073^*^
Non-striving	−0.047	0.195^**^	0.068	−0.283^**^	−0.008
Spaciousness	0.074^*^	0.233^**^	0.156^**^	−0.322^**^	0.089^*^
Acceptance	0.114^**^	0.378^**^	0.116^**^	−0.373^**^	0.043
Non-separation from others	0.158^**^	0.318^**^	0.142^**^	−0.313^**^	0.024
Revelation of truth	0.142^**^	0.226^**^	0.170^**^	−0.311^**^	0.018
Flow	0.099^**^	0.257^**^	0.138^**^	−0.325^**^	0.081^*^
Perfect as it is	0.082^*^	0.246^**^	0.090^*^	−0.339^**^	0.016
Openness	0.290^**^	0.287^**^	0.191^**^	−0.326^**^	0.113^**^
Appreciation	0.098^**^	0.197^**^	0.157^**^	−0.214^**^	0.125^**^
Same and different	0.157^**^	0.321^**^	0.140^**^	−0.240^**^	0.125^**^
Cronbach’s alpha	0.61	0.43	0.49	0.66	0.27

*p < 0.05 level (2-tailed).

**p < 0.01 level (2-tailed).

#### Self-reported nondual awareness

An additional type of convergent validity was tested by examining the relationship between oneness factor scores and participants’ own self-reported experience of non-dual awareness frequency and intensity (based on two different definitions of nondual awareness). When correlations of non-dual awareness frequencies with oneness factors from both definitions (all positive) were averaged (see Table 10), the overall mean was 0.400 and ranged from 0.252 (*safety and reassurance*) to 0.589 (*Part of a whole/connection with everything*, *POAW/CWE*).

Validity was also tested by comparing correlations with participants’ own direct subjective judgements based on responses to two questions reporting their *intensity* of experiencing nondual awareness across two definitions of non-dual experience (see Table 10). In all cases, correlations were positive and average correlations across the four measures were particularly strong for *Foundational Orientations* factors (*M* = 0.48). The overall mean correlation was *M* = 0.394, with *safety and reassurance* and *POAW/CWE* having the lowest (*M* = 0.244) and highest (*M* = 0.556) average correlations, respectively. Most (81.0%) average correlations were medium sized (0.3) or larger (Cohen, 1992). *Ego-dissolution* had an average correlation of 0.405.

**Table 10:** Bivariate spearman correlations between 21 oneness scale factors and direct nondual awareness questions.

Factor	Theme	Theme Number	Unified experience where you feel a sense of oneness _ How often? (Definition 1)	Unified experience in which usual subject/object not dominant _ How often? (Definition 2)	I have a deep experience of nondual awareness in my everyday life (Definition 1)	I have a profound recognition of nondual awareness in my everyday life (Definition 1)	I have a deep experience of nondual awareness in my everyday life (Definition 2)	I have a profound recognition of nondual awareness in my everyday life (Definition 2)
POAW/CWE	Foundational orientations	7	0.651^**^	0.527^**^	0.645^**^	0.658^**^	0.464^**^	0.456^**^
Ego-dissolution	Self-perception	3	0.336^**^	0.464^**^	0.374^**^	0.390^**^	0.436^**^	0.419^**^
Clarity	Deconditioning and embodiment	4	0.350^**^	0.275^**^	0.382^**^	0.366^**^	0.322^**^	0.323^**^
Nowness	Foundational orientations	7	0.413^**^	0.372^**^	0.410^**^	0.444^**^	0.341^**^	0.350^**^
Safety and reassurance	Emotions	6	0.309^**^	0.194^**^	0.320^**^	0.273^**^	0.197^**^	0.186^**^
Non-judgementality	Deconditioning and embodiment	4	0.326^**^	0.298^**^	0.327^**^	0.336^**^	0.302^**^	0.300^**^
Timelessness	Foundational orientations	7	0.427^**^	0.472^**^	0.419^**^	0.442^**^	0.442^**^	0.451^**^
Spontaneity	Action orientation	1	0.354^**^	0.406^**^	0.374^**^	0.400^**^	0.372^**^	0.400^**^
Peace	Emotions	6	0.499^**^	0.420^**^	0.515^**^	0.504^**^	0.404^**^	0.380^**^
Compassion	Interpersonal emotions	5	0.371^**^	0.282^**^	0.344^**^	0.344^**^	0.185^**^	0.224^**^
Self-compassion	Deconditioning and embodiment	4	0.414^**^	0.358^**^	0.433^**^	0.421^**^	0.318^**^	0.331^**^
Non-striving	Action orientation	1	0.263^**^	0.400^**^	0.304^**^	0.335^**^	0.436^**^	0.409^**^
Spaciousness	Foundational orientations	7	0.544^**^	0.539^**^	0.555^**^	0.558^**^	0.504^**^	0.494^**^
Acceptance	Deconditioning and embodiment	4	0.476^**^	0.396^**^	0.487^**^	0.488^**^	0.395^**^	0.404^**^
Non separation from others	Foundational orientations	7	0.561^**^	0.523^**^	0.591^**^	0.605^**^	0.472^**^	0.476^**^
Revelation of truth	Deconditioning and embodiment	4	0.490^**^	0.403^**^	0.496^**^	0.516^**^	0.412^**^	0.406^**^
Flow	Action orientation	1	0.457^**^	0.416^**^	0.470^**^	0.459^**^	0.376^**^	0.370^**^
Perfect as it is	Foundational orientations	7	0.428^**^	0.403^**^	0.443^**^	0.472^**^	0.414^**^	0.419^**^
Openness	Interpersonal emotions	5	0.340^**^	0.247^**^	0.311^**^	0.328^**^	0.222^**^	0.264^**^
Appreciation	Emotions	6	0.336^**^	0.274^**^	0.286^**^	0.286^**^	0.233^**^	0.239^**^
Same and different	Self-perception	3	0.439^**^	0.337^**^	0.432^**^	0.447^**^	0.286^**^	0.308^**^

*p < 0.05 level (2-tailed). **p < 0.01 level (2-tailed).

## Discussion

Following established guidelines in scale development (DeVellis, 2017), the current study set out to develop a new Oneness scale grounded in the experience of people who have experienced oneness. The Oneness scale is an 84-item measure composed of 21 factors (see Table 4), namely Part of a whole/Connection with everything, Ego-dissolution, Clarity, Nowness, Safety and reassurance, Non-judgementality, Timelessness, Spontaneity, Peace, Compassion, Self-compassion, Non-striving, Spaciousness, Acceptance, Non-separation from others, Revelation Of Truth, Flow, Perfect as it is, Openness, Appreciation, Same And Different. In line with past research, factors relating to affective, cognitive, motivational, and relational aspects of oneness were identified. The factors possess good internal consistency (*r*_p_ = 0.78–0.95).

Before discussing the implications of these findings, it is important to note that the present study is cross-sectional and observational. The associations reported between oneness factors, mindfulness, personality, and well-being cannot be used to infer causal or directional relationships. Where the discussion that follows draws on theoretical models to suggest possible mechanisms or pathways -- for example, the role of contemplative practice in fostering oneness experiences, or the potential links between particular oneness components and well-being -- these interpretations should be understood as tentative and hypothesis-generating. They are offered to contextualise the findings within the broader literature and to identify directions for future longitudinal and experimental research, not as claims directly supported by the current data.

### Content validity

Content validity evaluates how well a scale covers all relevant parts of the construct it aims to measure. In the current study, the oneness construct is constituted by the 7 overarching themes and 30 factors resulting from content expert consultation. The 30 components selected following expert consultation and the 21 factors extracted in factor analysis are presented in Supplementary Table S2. Notably, 6 of the original 7 themes (excluding *other perception*) are represented by the retained factors, including 100% (3/3) of *action* (T1) factors, 86% (6/7) of *foundational orientations* (T7) factors, and at least 60% of factors were retained in the remaining themes. The exception here is *other perception* (T3) where no factors were retained although one original T3 factor, *connection with everything*, was recategorized as part of the *Foundational Orientations* (T7) theme. Overall, this suggests that the oneness scale developed in the current study shows good construct coverage across the overarching theoretical oneness themes.

The construct coverage in the oneness scale can also be compared to components of oneness/nondual awareness identified in previous research and theory (see Supplementary Table S4). This comparison allows for further reflection on retained and unretained factors in the current study. A closer look at unretained factors in the current study suggests that some may be conceptually covered by the retained factors either singly or in combination. For example, *equanimity* may be covered by *peace and perfect as it is*. *Unconditional love and connection with others* may be covered by *non-separation from others, appreciation, and openness*. On the other hand, *aliveness*, while having similarities to *nowness*, is not as well captured by retained factors. At the same time, *aliveness* (‘a sense of alert aliveness’) appears to be conceptually similar to oneness components in earlier research such as, e.g., *intensified presence* [see, e.g., Taylor (2013)].

There are a number of proposed components of oneness/nondual awareness appearing in prior theory and research that were not well-represented in the initial 30 oneness components hypothesized in the current study. This includes components of oneness/nondual awareness more directly related to hedonic forms of wellbeing (e.g., bliss, ecstatic pleasure, spontaneous joy, contentment, positive affective states, deeply felt positive mood). In the case of *bliss*, there was an explicit decision in this study to exclude items measuring this factor, for psychometric and practical reasons (see introduction), particularly given the research objective of developing a scale that can be used to examine relations between oneness experience and wellbeing without confounding the two constructs in the scale development process. On the other hand, the factors *perfect as it is* and *non-striving* may capture elements of *bliss* or may be mechanisms through which subjective or hedonic wellbeing outcomes are experienced. Other components from earlier research not included in the current scale include *ineffability*. It is possible that the expert meditators who participated in the collective intelligence research informing construct development had sufficient understanding of their oneness/nondual awareness experiences and were thus able to articulate their experience – as such, *ineffability* was not highlighted by meditation experts. Other factors from previous research not included are *feelings of the holy*, *sacred or divine* – which may suggest that the meditation experts in the current scale development project were not generally adherents of theistic religions, where these feelings are more commonly expressed.

Some factors emerging in the current study also appear to capture somewhat unique properties that have received less attention in earlier research. For example, the *factor part of a whole/connection with everything* emphasizes a part/whole gestalt. Furthermore, *connection with everything* may be viewed as an absolute form of connectedness that is qualitatively different from more relative forms, such as feeling connected to particular people, groups, or aspects of the world.

The factor *connection with everything* was initially included under *other perception* (T2) as it was considered to be another type of connection like “connection with others”, and it was also considered different from constructs measuring *non-separation*. However, in the factor analyses *connection with everything* and *part of a whole* formed a single factor. This factor extraction makes sense if we notice the differences rather than similarities between *connection with everything* and other apparently similar factors such as *connection with others* [see also Leary et al., (2008)]. The difference between the construct *connection with everything* and *connection with others* is that the reference to everything is more absolute and thus alters the meaning of *connection* as used here. When one is connected to everything, there is nothing that one is not connected to. When there is nothing one is not connected to, being connected to something different from oneself is also meaningless, because difference from oneself can no longer be established through the appearance of disconnection. When seen in this way, there is now very little meaningful difference between being *part of a whole* and being *connected to everything* and hence it is understandable that these factors, which were originally hypothesized to be distinct, were extracted as one factor, and that this factor is appropriately categorised under *Foundational Orientations* theme (T7).

### Convergent validity

Evidence of convergent validity was of four types – convergent validity with self-report measures of nondual awareness, convergent validity with a hypothesised outcome of oneness (i.e., wellbeing), convergent validity with a hypothesised predictor of oneness (i.e., mindfulness), and convergent validity with personality traits expected to correlate with oneness (i.e., neuroticism and openness). In all four cases there was evidence of convergent validity.

### Convergent validity with self-report measures of non-dual awareness

Self-report of non-dual awareness was measured using questions evaluating frequency and intensity of experiences. Medium correlations with most factors and significant correlations across all, provide evidence of convergent validity for the oneness scale developed in the current study. The fact that more *foundational orientation* variables are found among the highest correlations may suggest that these ratings of nondual awareness reflect awareness of foundational aspects of oneness experience.

### Convergent validity with wellbeing measures

Convergent validity was also assessed by examining correlations between oneness factors and wellbeing factors, specifically subjective, psychological, and social wellbeing scores, as measured using the Mental Health Continuum (Short Form; MHC-SF) wellbeing scale (Lamers et al., 2010). Results suggest positive associations with all oneness factors (except for ego-dissolution) with 76% of these correlations having at least medium effect sizes. In general, the factors that were most obviously conceptually associated with wellbeing (e.g., *peace*, *appreciation*, *self-compassion*) are associated most strongly with wellbeing scale scores. These relationships are discussed in more detail below, focusing on each oneness theme in turn.

It is important to acknowledge that several oneness factors, including Peace, Self-Compassion, and Appreciation, share conceptual ground with the well-being constructs used for convergent validation in this study. This raises the question of whether positive associations between these particular factors and well-being scores reflect genuine convergent validity. In the context of the Oneness Experience Scale, these factors are intended to capture specific experiential qualities of oneness, for example, the sense of peace experienced as an intrinsic quality of oneness awareness, rather than peace as a general mood state or trait. Emerging theoretical work suggests that affective states experienced within the context of oneness or nondual awareness may be qualitatively distinct from their counterparts outside this context—for instance, experienced as more unconditional, stable, or spontaneous rather than dependent on external conditions [see, e.g., Dambrun et al. (2012) and Jones (2023)]. However, the present data cannot empirically distinguish between these possibilities, and this remains an important question for future research, ideally using discriminant validity analyses or experimental designs that can examine whether oneness-related affective states behave differently from non-oneness-related equivalents.

#### Foundational orientations [T7]

Earlier literature on oneness/nondual experience suggests that fundamental shifts in perception related to self, time, and space are central to this experience. As noted, 6 of 7 T7 factors were retained after factor analysis in the current study. It was predicted that higher scores on these factors would be associated with higher wellbeing, as these shifts in perception (e.g., being part of a whole, non-separation from others) may be positively associated with wellbeing. This hypothesis was confirmed, and the findings of the current study are also partially supported by an earlier study focused on measuring interconnectedness experiences and wellbeing (Yu et al., 2020). However, it should be noted that there were no corresponding scales available in the literature that measured other factors identified in the current study, specifically, *part of a whole*, *nowness*, *timelessness*, *spaciousness*, and *perfect as it is*. As such, the current study adds to the literature in identifying a positive relationship between *Foundational orientations* factors and wellbeing outcomes.

#### Emotions [T6]

Consistent with previous theory (e.g., Blackstone, 2012; Josipovic, 2021) and research (e.g., Koenig, 2008) suggesting that spiritual experience (and associated measures) often include wellbeing factors, three of the five hypothesized factors in T6, *Emotions*, were picked up in factor analysis, specifically, *safety and reassurance*, *peace* and *appreciation*, all of which have positive relationships with wellbeing in previous studies (Helliwell and Wang, 2018; Soysa et al., 2021; Kreitzer et al., 2009; Portocarrero et al., 2020). One factor that was not included was *equanimity* – a sense of peace regardless of what is happening, which has been considered a factor associated with oneness and wellbeing in previous studies (e.g., Chan et al., 2014). It is possible that the initial inclusion of two very similar factors, *peace* and *equanimity*, may have led to one of these factors being excluded statistically as part of the factor analysis process.

#### Interpersonal emotions [T5]

Two out of three interpersonal factors were retained – *openness* and *compassion* – both of which were associated with all wellbeing components, as predicted based on previous research [Compassion: Kirby et al. (2017); Openness: Sutin (2017)]. *Unconditional love* did not emerge as a factor in the factor analysis, possibly because the items on this factor were captured by other constructs (e.g., *non-separation from others*, *perfect as it is*, *appreciation*, *compassion*).

#### Deconditioning and embodiment [T4]

Five of seven deconditioning and embodiment theme factors were retained during factor analysis, *clarity*, *non-judgementality*, *self-compassion*, *acceptance*, and *revelation of truth*, and were associated with wellbeing as predicted based on earlier studies (e.g., Dickhäuser and Garbade, 2015; Ford et al., 2018; Hanley and Garland, 2019; Yaden Le Nguyen et al., 2017; Wahbeh et al., 2022). The two remaining factors aliveness and sensitivity, were not retained in the process of factor analysis. It is possible that variance associated with these factors was captured by selected factors, e.g., *aliveness resembles nowness*, and *sensitivity resembles openness*; the last of each pair was selected in the EFA.

#### Self-perception change [T3]

Two of three factors in this theme were retained, with the factor *being everything* excluded. As predicted, *ego-dissolution* – the only factor predicted to have a low positive or negative or non-significant correlation – had a non-positive, non-significant correlation with wellbeing. This suggests that ego-dissolution, at least for some participants, is not always a positive experience associated with wellbeing, and that wellbeing is more strongly associated with the unity-based or *relational* (Yaden Haidt et al., 2017) aspects of oneness (e.g., *part of a whole/connection with everything*, *non-separation from others*). It is also possible that ego-dissolution is not coterminous with other aspects of oneness, in the sense that it may precede (e.g., McCormick, 2010) or come after other oneness experiences [a possible exception being in the context of psychedelic experience, see, e.g., Nour et al. (2016)].

#### Other perception [T2]

While one T2 factor was retained in the factor analysis (*connection with everything*), it was statistically combined with another factor *part of a whole* in the Foundational orientations theme (see T7 above), arguably causing this factor to change theme and disappear as a distinct component. On the other hand, the non-selection of *connection with others* may be less surprising here. Arguably, a core theme of oneness and nondual awareness is disappearance of the experience of *other*.

#### Action orientation [T1]

All three action orientation items – *flow*, *non-striving* and *spontaneity*, were retained and, as hypothesized based on previous research [see Collins et al. (2009), Dambrun (2017), and Kipper and Shemer (2006), respectively], these factors were associated with wellbeing. The somewhat lower correlations with *non-striving* (range = 0.118 – 0.206) compared to other oneness factors (*M* = 0.357) may be explained by its possible associations with depression for some people who may not yet experience higher levels of oneness. While *non-striving* may suggest a more passive stance on reality, this is countered with *spontaneity* of actions and experiences of *flow* which imply engagement with reality and experience.

### Convergent validity with mindfulness measures

Convergent validity was also examined in proposed relationships with mindfulness. All five facets of the FFMQ were, on average, weakly to moderately correlated with oneness factors. Previous research (e.g., Tran et al., 2014) suggests that non-reacting and observing are sensitive to meditation practice [see also, Baer et al. (2008), Lilja et al. (2012), and Soler et al. (2014)]. Moreover, non-react may have different meanings among meditators and non-meditators (Tran et al., 2014). It is possible that meditators who increase in their powers of observation shift into greater non-reactivity and are more likely to experience oneness. Another possibility is that those who experience oneness develop the mindfulness features of more experienced meditators.

### Convergent validity with personality

Personality potentially represents another area where convergent validity can be examined. While positive correlations with extraversion, agreeableness, and conscientiousness and oneness-related variables (in contexts of meditation, mystical experience, or psychedelics) have been reported in earlier research [see Erritzoe et al. (2018), Hanley et al. (2018), Levenson et al. (2005), Netzband et al. (2020), and Weiss et al. (2023)] it is correlations with neuroticism and openness where the largest and most enduring correlations have been seen [see also Carhart-Harris et al. (2016) and McCulloch et al. (2022)]. In the current study, correlations for neuroticism, extraversion, agreeableness, and conscientiousness are broadly consistent with previous research, whereas those for openness are lower. In the case of Neuroticism (*α* = 0.66), we see consistently negative correlations with oneness factors. While neuroticism is associated with oneness experience, it is also plausible that changes in neuroticism represent a possible mechanism underlying the association between oneness and wellbeing. However, the directionality of these relationships cannot be established from the present data.

The correlations between oneness factors and personality traits should be interpreted in light of the low internal consistency of several BFI-10 subscales, as discussed in the Limitations section. Correlations involving openness, agreeableness, and conscientiousness in particular may be substantially attenuated by measurement unreliability. It is not immediately clear why internal consistency was lower in this dataset, given adequate performance reported previously (Rammstedt and John, 2007).

### Higher-order factor structure

In addition to the 21-factor scale, a higher-order confirmatory factor analysis was conducted to examine whether the first-order factors could be organised into a smaller number of broader dimensions. The analysis yielded a six-factor higher-order structure—Foundations, Self, Deconditioning, Emotions, Action, and Interpersonal—which corresponds to the overarching thematic structure identified in prior qualitative research (Van Lente and Hogan, 2020). The higher-order model demonstrated acceptable fit, and the majority of first-order factor loadings exceeded 0.60. This structure offers a more parsimonious framework for researchers who seek to measure oneness experience across domain levels, while the full 21-factor version remains available when fine-grained differentiation of oneness components is required. This multi-level measurement approach is broadly consistent with other multidimensional instruments in psychology that offer both detailed and summary-level scoring (e.g., the facets and domains of the NEO-PI-R; Costa and McCrae, 1992). However, as with the first-order CFA, the higher-order results were obtained using the same sample and await confirmation in an independent sample.

### A focus on ego-dissolution

After the factor part of a *whole/connection with everything* (34%), *ego-dissolution* (7%) explains the second highest amount of variance in the oneness construct. In some theoretical and empirical approaches, ego-dissolution is seen as part of a connection or unity factor, e.g., as measured in the ego-dissolution scale (Nour et al., 2016) and the Hood mysticism scale (Hood, 1975). Although the two factors are correlated, the current study suggests that it may be possible to feel part of a whole without the sense of ego dissolving, and vice versa. This is consistent with a number of religious and theoretical approaches and empirical findings where this distinction is found, including Buddhism (ego-dissolution /non-self; e.g., Olendzki, 2006, Ramm, 2021), the distinction between extrovertive (unity-related) vs. introvertive (ego-dissolution related) components of mystical experience (Stace, 1960), as well as relational and annihilational components of mystical experience (Yaden Haidt et al., 2017).

Also, in the current study, ego-dissolution uniquely does not correlate significantly with any of the wellbeing variables. Two observations will be made that may explain this finding. First, accounts of ego-dissolution in many religions equate it with a challenging experience, or a type of ‘death’ including terms such ‘Great death’ in Zen Buddhism (Safran, 2012) or fana (‘annihilation’) in Muslim Sufism (Encyclopaedia Britanica, 2023) and it also appears in Christianity (Merkur, 2007), suggesting that this ‘death-like’ experience is historically and culturally pervasive. One Buddhist scholar suggests that the symbolic ‘death’ or the fear of the death of the ‘ego’ can be experienced as similar to the actual physical threat of death (Loy, 2000). In general, spirituality and psychological practices deriving from spirituality seem to support practices which involve approaching uncomfortable or fearful thoughts via meditation or daily life practice (Hayes-Skelton and Eustis, 2020) and coming to accept them, relax, or surrender in the face of them (Singer, 2007). *Experiential approach* (the opposite of *experiential avoidance*) has been considered a putative mechanism of change for psychedelics. In Zeifman et al. (2023) study, reductions in anxiety occurred via reductions in experiential avoidance. Moreover, ego-dissolution and psychological insight also predicted less experiential avoidance after psylocibin therapy. Ego-threats are often associated with anxiety (Leary et al., 2009) but mindfulness is also associated with reduced reactivity to ego-threats (Brown et al., 2016; Brown and Leary, 2016).

Another possibility is that ego-dissolution may not have been measured with adequate (content) validity in this project. Notably, a limited number of items were provided by and confirmed by experts. Three of four ego-dissolution items include the term “self” coupled with the word ‘dissolve’. It is possible that these particular items may correlate non-positively with wellbeing. Also, ego-dissolution is arguably a much broader concept than that mentioned here and to understand its relationship with wellbeing it will be necessary to measure ego-dissolution more precisely. Forms of dissolution of ego seen in religious, spiritual, psychedelic, and mystical experiences appear to have the experience of ‘not feeling separate’ at its core (Lindström et al., 2022). Another aspect of ego-dissolution includes possible changes in motivation and focus of attention, as egotistical, egocentric and egoic motivations are dissolved (Leary and Diebels, 2017), which might allow people to face suppressed aspects of self with non-judgement, acceptance, and self-compassion (Costeines, 2009). Understood and measured in this way, ego-dissolution might emerge as more positively related to wellbeing outcomes. Future studies will be important to investigate these issues further.

## Limitations

There are a number of limitations to this study. First, the data is cross-sectional and thus cannot be used to make any causal claims. All interpretations in the Discussion regarding possible mechanisms or pathways linking oneness, mindfulness, personality, and well-being should be considered speculative and hypothesis-generating rather than explanatory.

Second, the exploratory and confirmatory factor analyses were conducted using the same sample, which increases the risk of overfitting and may produce upwardly biased estimates of model fit (Fokkema and Greiff, 2017). Some features of the study design may partially offset this concern: factor retention in the EFA was determined using Horn’s parallel analysis (Horn, 1965), which is widely regarded as one of the more accurate methods for determining the number of factors to retain (e.g., Zwick and Velicer, 1986; Hayton et al., 2004); the resulting factor structure showed considerable correspondence with an *a priori* conceptual model developed independently through prior collective intelligence research; and Fokkema and Greiff (2017) note that the risk of overfitting tends to decrease with increasing sample size. Nevertheless, these considerations reduce rather than eliminate the limitation, and the CFA results—both first-order and higher-order—should be considered preliminary. Replication with an independent sample is needed to confirm the stability and generalisability of the proposed factor structures.

Third, although items were randomised within their factor groupings in the online survey, the decision to present similar items together, which was adopted to reduce respondent burden, may have had the effect of increasing within-factor correlations, potentially inflating the number of factors identified (Siminski, 2008). A future study presenting items in a fully randomised order would help to clarify whether a similar factor structure is obtained.

Fourth, a forced answering approach was used (all questions were non-optional) to reduce participant attrition and simplify analysis; however, this may have bothered some participants who would have preferred not to answer certain questions, and it may have increased noise in the data. Fifth, no test questions were included to monitor participant attentiveness; low attentiveness may also have contributed to noise in the data (Ward and Meade, 2023; Braitman et al., 2022).

Sixth, the brief personality measure used (BFI-10), while minimising participant burden in an already lengthy survey, yielded low internal consistency for several traits, particularly Openness (*α* ≈ 0.27), Agreeableness (*α* ≈ 0.43), and Conscientiousness (*α* ≈ 0.49), which substantially limits the interpretability of personality-related correlations for these traits. Correlations involving Neuroticism (*α* ≈ 0.66) and Extraversion (*α* ≈ 0.61) may be considered more interpretable (DeVellis, 2017), though any interpretation still requires caution given the two-item measurement for each personality construct.

Seventh, many participants were recruited from meditation-related communities and through self-selected online samples, which introduces potential selection bias and likely overrepresentation of individuals with meditation experience and oneness experiences. This may have inflated associations with mindfulness and restricted variance in ways that limit generalisability to the broader population.

Finally, while the 21-factor structure is large and highlights concerns regarding parsimony, the scale was developed with the deliberate aim of capturing the full breadth of oneness experience as reported by experiencers, rather than imposing a priori constraints on the construct’s dimensionality. The higher-order factor analysis yielding a six-factor structure offers a more parsimonious alternative for applied research, though these results too should be considered preliminary. As the construct of oneness experience becomes better understood, further refinement of the scale—including potential reduction of items and factors—will be an important direction for future research.

## Conclusion

Following established guidelines in scale development (DeVellis, 2017) and in psychometric statistical analysis (Costello and Osborne, 2005; Worthington and Whittaker, 2006; Byrne, 2010), a new measure of Oneness experience has been developed. In developing this measure, meditation experts who had experienced oneness were consulted directly in a collective intelligence inquiry of oneness self-perceptions (Van Lente and Hogan, 2020). Taking this step was consistent with a mixed-methods approach to scale construction (Padgett, 1998) which advises qualitative exploration of concepts preceding quantitative work, resulting in greater validity as constructs and associated scale items are derived from real life experience (Rowan and Wulff, 2007). Exploratory structural equation modelling (ESEM) was used to confirm the factor structure identified using exploratory factor analysis (EFA). This resulted in a scale with 84 items measuring 21 factors (See Table 4 for psychometric properties and Supplementary Table S3 for the complete scale and administration instructions). Other researchers may wish to develop oneness scales based on the wider set of constructs uncovered in the current study. The full set of items are provided in Supplementary Table S2. There may be alternative models which fit the data equally well and it is possible that alternative models could arise if alternative criteria had been adopted during the factor analysis process. The Oneness Scale is a measure suited for research on oneness as an ongoing experience. Further psychometric investigation is needed to establish the predictive, discriminant and known groups validity.

## Data Availability

The raw data supporting the conclusions of this article will be made available by the authors, without undue reservation.

## Footnotes

1 Although Worthington and Whittaker (2006) criteria suggest 0.4 as a minimum for loadings, a minimum of 0.5 was used for this group of items, as they are highly correlated. This was necessary to reduce the number of items (and factors) more substantially in the final scale.
