# Supplementary material for: Development and psychometric evaluation of the Oneness Experience Scale
Source: Front Psychol. 2026 Aug 3;17:1789746. doi: 10.3389/fpsyg.2026.1789746 (PMC13478011; doi:10.3389/fpsyg.2026.1789746)
Supplement: Supplementary file 1 [file Supplementary_File_1.pdf]

Supplementary Table S1: *Conceptualisations of components of temporary mystical experience, permanent wakefulness, and permanent nondual consciousness (oneness) from various researchers and theorists, in order of frequency of endorsement*

**PART 1**<br>**Focus is on <u>temporary/transient</u> mystical or enlightenment/spiritual and transformative experiences that are 'temporary'**<br>**(Qualitative research)**			**PART 2**<br>**Focus is on <u>permanent</u> wakefulness**<br>**(Quantitative research)**	**PART 3**<br>**Focus is on <u>permanent</u> nondual consciousness**<br>**(Qualitative research)**		**PART 4**<br>***Theoretical* features of permanent nondual awareness (based on a review of Asian traditions)**
**James (1902/2002) ("Religious Experience")**	**Stace (1960) / Pahnke (1966) (P – Pahnke only) ("Mystical experience")**	**Newberg and Waldman (2017) ("enlightenment" [a]) Includes direct experiences and effects, which endure after experience has stopped**	**Kilrea, Taylor, Bilodeau, Wittmann, Linares Gutiérrez & Kübel (2023) ("Wakefulness") (Table 1)**	**Costeines (2009) [b] ("Nondual consciousness")**	**Fire (2011) [c] ("Nondual consciousness")**	**Josipovic (2021) ("Nondual awareness")**
Oneness	Oneness (2 types): 1 Extrovertive – "all things are one", inner subjectivity in all things, 2 Introvertive–- unitary pure consciousness /void, nonspatial/non-temporal	A sense of *unity* or connectedness	Sense of Connection	Nondual ontology (direct knowing that everything is one consciousness)	Felt sense of No Division (also described as a sense of oneness). Felt sense of Form and formlessness (interchanging form and formlessness, emptiness/ fullness, Non-localised)	Emptiness/ Nonduality, Singularity or unity// No self/self, Boundless, timeless spaciousness
Passivity, no control		A sense of surrender of loss of voluntary control.	Attitude of acceptance/letting go/surrender	Nondual action	More letting go and allowing	Bliss (without intention)
	Joy, happiness / [P: deeply felt positive mood]	Effect: Happier and more contented with life		Spontaneous joy	Positively experienced	Bliss/Ecstatic pleasure
	Blessedness, peace / [P: deeply felt positive mood]	Effect: Happier and more contented with life		Beatific Peace, Positive cosmology	Positively experienced	Bliss
	Blessedness, peace / deeply felt positive mood	An incredible intensity of experience	Intensified perception		Energy, Dynamic	Ecstatic pleasure
	Transcendence of time or space		Timelessness	Timeless awareness	Nonlocalized	Boundless, timeless spaciousness
		Effect: more open-minded	Authenticity		More letting go and allowing, Felt sense of openness	Boundless, timeless spaciousness
	Objectivity or reality	A sense of clarity and *new understanding* in a fundamental way // Effect: more open-minded		Mental lucidity	More clarity	

Noetic quality (New spiritual insights)	Objectivity or reality [P: a feeling of insight or illumination]	A sense of clarity and *new understanding* in a fundamental way // Effect: more open-minded			Felt sense of truth	
		An incredible intensity of experience	Increased presentness / Intensified perception			Being or presence
Ineffability	(Alleged) Ineffability				Ineffable	
			Increased inner security	Awareness of spiritual immortality	More trust	
			Authenticity	Nondual action	Felt sense of a bigger underlying movement [Spontaneous]	
	Transcendence of time or space				Non-localised, Felt sense of openness	Boundless, timeless spaciousness
			Increased presentness			Being or Presence/ Boundless, timeless spaciousness
	Dissolution of individuality (introvertive mysticism)					Emptiness// No self/self
		Effect: don't ruminate on past mistakes	Attitude of acceptance/letting go/ surrender			
				Nondual action	More ease	
	Paradoxicality					No self/self// Emptiness/ Nonduality
			Reduced identification with thoughts and mental constructs	Disidentification from mental constructs		
	Feeling of the holy, sacred, or divine					
		Effect: Not as worried or nervous about facing and solving problems				
			Reduced judgemental attitude			
			Changed attitude to body			
			Intuitive decision-making			
			Transpersonal sense of purpose			
			Decreased sense of group identity			

			Reduced Cognitive Activity			
				Awareness of spiritual immortality		
				Absence of neurotic suffering		
				Unitive relationships		
				Unitive love		
					Verticality	
					Felt sense of "Me-ness"	
						Luminosity or radiance
						Non-conceptual reflexivity

Note. [a] Newberg and Waldman (2017) also discuss a 'big E' 'Enlightenment' which is considered to be permanent. However, this has not been included in this table.
[b] The terms in this column are themes from the section *Traits of nondual consciousness* (p.116)
[c] The terms in this column are themes from three sub-categories created in the thematic analysis: Primary category 1: Presence of nondual consciousness in one's life/ Subcategory 5: *Impact of nondual consciousness on life*, p. 146); Primary category 2: Directly felt experience of nondual consciousness itself, Subcategory 1: *Essential qualities of the felt experience of nondual consciousness* (p. 160) and Subcategory 2: *Felt Sense of Nondual Consciousness* (p. 168).

Supplementary Table S2: *All 179 Items including those selected in EFA, classified by theme, category, hypothesized and EFA factors. Factor loadings, R2, factor reliabilities, means and standard deviations reported for items selected in EFA.*

**Theme (with number)**	**Category**	**Hypothesised Factor**	**Factor number**	**Actual Factor from EFA**	**Hypothesised Items and Items selected through EFA and Item reduction**	**Item/ Factor Loading**	**R^2**	**Cronbach's Alpha**	***Mean (SD)***
7 Foundational orientations	Wholeness	Wholeness	1	Connection With Everything	A feeling of connection to everything*	0.89	0.78	0.94	3.54 (1.07)
7 Foundational orientations (was Theme 2 –- Other perception)	Connectedness	Connection with everything (4)		Part Of A Whole	Feeling that I am part of a greater unity or oneness*	0.87	0.75	0.94	3.54 (1.07)
				Connection With Everything	Experiencing connection with all that is*	0.82	0.67	0.94	3.54 (1.07)
				Part Of A Whole	Feeling that my individual life is part of a greater whole*	0.81	0.66	0.94	3.54 (1.07)
				Part Of A Whole	A sense of being an intrinsic part of all that is*	0.8	0.64	0.94	3.54 (1.07)
				Connection With Everything	Feeling a sense of connection to everyone and everything*	0.77	0.59	0.94	3.54 (1.07)
				Connection With Everything	Feeling connected with all of nature*	0.73	0.53	0.94	3.54 (1.07)
				Part Of A Whole	A sense that I am part of an all-pervading consciousness*	0.72	0.51	0.94	3.54 (1.07)
				Connection With Everything	A sense of connection between me and the universe				
				Part Of A Whole	A sense of being a wave that is always part of the sea				
				Part Of A Whole	Feeling that I am part of everything				
3 Self-perception	Identity perception change	Ego dissolution (4)	2	Ego-dissolution	A feeling that my self is dissolving*	0.96	0.92	0.95	2.56 (1.26)
				Ego-dissolution	A sense of me dissolving*	0.91	0.82	0.95	2.56 (1.26)
				Ego-dissolution	A sense of dissolution of the self*	0.91	0.82	0.95	2.56 (1.26)
				Ego-dissolution	A sense of my separate identity being dissolved away*	0.82	0.67	0.95	2.56 (1.26)
				Ego-dissolution	A sense of dissolution of my ego				
				Ego-dissolution	Feeling that there is no "I"				
4 Deconditioning and Embodiment	Clarity	Clarity (4)	3	Clarity	A feeling of clarity*	0.94	0.87	0.92	3.57 (0.99)
				Clarity	A sense of clarity*	0.93	0.86	0.92	3.57 (0.99)
				Clarity	Seeing things clearly*	0.78	0.6	0.92	3.57 (0.99)
				Clarity	Everything is perceived with clarity*	0.76	0.58	0.92	3.57 (0.99)

				Clarity	Seeing reality without distortion				
				Clarity	Thinking in a way that is clear and penetrating				
7 Foundational orientations	Time-perception change	Nowness	4	Nowness	Being here in the present*	0.91	0.83	0.92	3.8 (1.01)
				Nowness	A sense of being present in this moment*	0.85	0.72	0.92	3.8 (1.01)
				Nowness	Being in the moment*	0.85	0.72	0.92	3.8 (1.01)
				Nowness	A sense of being focused on the present*	0.74	0.55	0.92	3.8 (1.01)
				Nowness	A sense of eternal nowness				
				Nowness	My attention is fully in the now				
6 Emotions	Trust	Safety and Reassurance	5	Safety And Reassurance	A sense of being taken care of*	0.85	0.72	0.88	3.94 (0.93)
				Safety And Reassurance	A sense of safety and reassurance*	0.77	0.59	0.88	3.94 (0.93)
				Safety And Reassurance	Feeling safe*	0.76	0.58	0.88	3.94 (0.93)
				Safety And Reassurance	A feeling of support and love*	0.75	0.56	0.88	3.94 (0.93)
				Safety And Reassurance	A sense of reassurance				
				Safety And Reassurance	A sense of trust				
4 Deconditioning and Embodiment	Acceptance	Non-judgementality	6	Non-judgementality	No longer being judgemental of others*	0.93	0.86	0.89	3.64 (1)
				Non-judgementality	No longer judging others behaviour*	0.87	0.75	0.89	3.64 (1)
				Non-judgementality	No longer stereotyping others*	0.8	0.65	0.89	3.64 (1)
				Non-judgementality	Feeling less judgemental to others*	0.68	0.46	0.89	3.64 (1)
				Non-judgementality	A sense of forgiveness towards self and others				
				Non-judgementality	Experiencing judgement-free thought				
7 Foundational orientations	Time-perception change	Timelessness	7	Timelessness	A sense of being timeless*	0.86	0.74	0.9	2.82 (1.24)
				Timelessness	A sense of timelessness*	0.85	0.72	0.9	2.82 (1.24)
				Timelessness	A sense of being outside of time*	0.84	0.71	0.9	2.82 (1.24)
				Timelessness	A sense of there being no time*	0.71	0.51	0.9	2.82 (1.24)
				Timelessness	A sense that time is not real at the deepest level				

				Timelessness	A sense of time being meaningless					
1 Action orientation	Free-flow	Spontaneity	8	Spontaneity	A sense of my actions happening spontaneously*	0.86	0.74	0.84	2.82 (1.24)	
				Spontaneity	A sense of spontaneity in my actions*	0.85	0.72	0.84	2.82 (1.24)	
				Spontaneity	My spontaneous actions feel natural*	0.61	0.37	0.84	2.82 (1.24)	
				Spontaneity	A sense that my spontaneous actions do not need to be controlled*	0.58	0.33	0.84	2.82 (1.24)	
				Spontaneity	A sense that everything happens on its own					
				Spontaneity	Thoughts come and go freely					
6 Emotions	Unconditional peace	Unconditional Peace	9	Peace	Feeling tranquil*	0.9	0.81	0.94	3.48 (1.11)	
				Peace	Feelings of peace and calm*	0.88	0.77	0.94	3.48 (1.11)	
				Peace	A strong sense of peace and calm*	0.87	0.76	0.94	3.48 (1.11)	
				Peace	Feeling serene*	0.87	0.76	0.94	3.48 (1.11)	
				Peace	A feeling of deep peace					
				Peace	A feeling of inner stillness					
5 Interpersonal emotions	Open-heartedness	Compassion (4)	10	Compassion	A feeling of great compassion*	0.85	0.73	0.84	4.39 (0.67)	
				Compassion	Feelings of empathy and compassion*	0.82	0.68	0.84	4.39 (0.67)	
				Compassion	Feeling great warmth towards others*	0.73	0.53	0.84	4.39 (0.67)	
				Compassion	A happiness for others being happy*	0.59	0.35	0.84	4.39 (0.67)	
				Compassion	Having great patience with others					
				Compassion	Wanting others to be happy					
4 Deconditioning and Embodiment	Acceptance	Self-compassion (4)	11	Self-compassion	Loving myself the way I am*	0.87	0.76	0.9	3.73 (1.07)	
				Self-compassion	A deep acceptance and love for what I am*	0.81	0.65	0.9	3.73 (1.07)	
				Self-compassion	Acceptance of my personality*	0.78	0.61	0.9	3.73 (1.07)	
				Self-compassion	A feeling of self-compassion*	0.74	0.55	0.9	3.73 (1.07)	
				Self-compassion	A feeling of complete acceptance of where I am in life					
				Self-compassion	Feeling patient with myself					

1 Action orientation	Free-flow	Non-striving (4)	12	Non-striving	No longer striving*	0.89	0.78	0.82	2.73 (1.12)
				Non-striving	A sense of no longer having goals*	0.81	0.66	0.82	2.73 (1.12)
				Non-striving	No longer searching for meaning*	0.62	0.39	0.82	2.73 (1.12)
				Non-striving	Not forcing things to go my way*	0.55	0.3	0.82	2.73 (1.12)
				Non-striving	A feeling of no longer struggling				
				Non-striving	No longer needing to control				
7 Foundational orientations	Unboundedness	Spaciousness (4)	13	Spaciousness	A sense of limitless space*	0.88	0.77	0.92	3.06 (1.22)
				Spaciousness	A sense of open, infinite space*	0.85	0.71	0.92	3.06 (1.22)
				Spaciousness	A sense of being boundless*	0.81	0.66	0.92	3.06 (1.22)
				Spaciousness	Experiences of boundarylessness*	0.81	0.65	0.92	3.06 (1.22)
				Spaciousness	A sense of being infinite				
				Spaciousness	Experiences of spaciousness				
4 Deconditioning and Embodiment	Acceptance	Acceptance (4)	14	Acceptance	Accepting everything as it is*	0.88	0.77	0.9	3.44 (1.11)
				Acceptance	A deep sense of acceptance no matter how good or how difficult the situation*	0.81	0.66	0.9	3.44 (1.11)
				Acceptance	Allowing everything to be as it is*	0.79	0.63	0.9	3.44 (1.11)
				Acceptance	A deep sense of acceptance*	0.77	0.59	0.9	3.44 (1.11)
				Acceptance	A sense of there being space for everything				
				Acceptance	Having an attitude of acceptance				
7 Foundational orientations	Unboundedness	Non-separation from others (4)	15	Non Separation from Others	A sense of not being separate from others*	0.94	0.88	0.89	3.19 (1.1)
				Non Separation from Others	No longer feeling separate from others*	0.83	0.69	0.89	3.19 (1.1)
				Non Separation from Others	Knowing that I am ultimately not separate from others*	0.72	0.52	0.89	3.19 (1.1)
				Non Separation from Others	A disappearance of boundaries between self and other*	0.71	0.5	0.89	3.19 (1.1)
				Non Separation from Others	A sense of barriers dissolving between myself and others				
				Non Separation from Others	Seeing that I am not separate from others at the deepest level				
4 Deconditioning and Embodiment	Clarity	Revelation of truth (4)	16	Revelation Of Truth	Realising ultimate truths*	0.88	0.78	0.88	3.33 (1.1)

				Revelation Of Truth	A sense that the core nature of things has been revealed*	0.78	0.61	0.88	3.33 (1.1)
				Revelation Of Truth	Seeing the essential nature of reality*	0.77	0.59	0.88	3.33 (1.1)
				Revelation Of Truth	Realising who I am at the deepest level*	0.6	0.35	0.88	3.33 (1.1)
				Revelation Of Truth	A sense of having realised my true self				
				Revelation Of Truth	A sense of having seen something that is central to all existence				
1 Action orientation	Free-flow	Flow (4)	17	Flow	A feeling of flow*	0.84	0.71	0.89	3.6 (1)
				Flow	A feeling of life flowing freely*	0.81	0.65	0.89	3.6 (1)
				Flow	A feeling of life flowing spontaneously*	0.74	0.55	0.89	3.6 (1)
				Flow	A sense of flow even in the face of resistance*	0.72	0.52	0.89	3.6 (1)
				Flow	A sense that things just seem to happen spontaneously				
				Flow	Not being concerned with how others might be evaluating my free-flowing spontaneous action				
7 Foundational orientations	Wholeness	Perfect as it is (3)	18	Perfect As It Is	A recognition that everything is perfect*	0.89	0.78	0.92	2.46 (1.3)
				Perfect As It Is	Feeling that the world is perfect as it is*	0.82	0.67	0.92	2.46 (1.3)
				Perfect As It Is	A sense that everything is already perfect and complete just as it is*	0.8	0.64	0.92	2.46 (1.3)
				Perfect As It Is	A sense of being enough as I am				
				Perfect As It Is	Feeling complete as I am				
				Perfect As It Is	Realizing that everything works out as it should				
5 Interpersonal emotions	Open-heartedness	Openness (3)	19	Openness	Communicating openly*	0.83	0.69	0.81	4.11 (0.89)
				Openness	Feelings of openness*	0.74	0.54	0.81	4.11 (0.89)
				Openness	Being open to feel my feelings*	0.71	0.5	0.81	4.11 (0.89)
				Openness	Being open to all experiences				
				Openness	Embracing what is				
				Openness	Being open to uncertainty				
6 Emotions	Appreciation	Appreciation (3)	20	Appreciation	Appreciating contentment and peace*	0.78	0.61	0.78	4.52 (0.65)
				Appreciation	Savouring moments of quiet and silence*	0.73	0.54	0.78	4.52 (0.65)
				Appreciation	A feeling of appreciating simple things*	0.63	0.4	0.78	4.52 (0.65)

				Appreciation	A sense of gratitude				
				Appreciation	A sense that things are new and interesting				
				Appreciation	Appreciating others				
3 Self-perception	Identity perception change	Same but different (3)	21	Same And Different	A sense of being the same despite our differences*	0.8	0.64	0.82	3.98 (0.91)
				Same And Different	A sense of commonality underneath our differences*	0.79	0.63	0.82	3.98 (0.91)
				Same And Different	Feeling one with others despite differences*	0.54	0.3	0.82	3.98 (0.91)
				Same And Different	A sense of being something and nothing at the same time				
				Same And Different	Feeling simultaneously unique and part of the collective				
				Same And Different	A sense that we are all unique in expression but the same in essence				
**Factors below were not selected during EFA item reduction**									
3 Self-perception	Identity perception change	Being everything		Being Everything	A sense of seeing oneself as everything				
				Being Everything	A sense that there is no longer a perceived duality of the observer and the observed				
				Being Everything	A sense that I am this moment				
				Being Everything	A sense of being nothing and everything at the same time				
				Being Everything	A sense of being the totality of life				
				Being Everything	Being everything in this moment				
2 Other perception	Connectedness	Connection with others		Connection With Others	Experiencing a feeling of connectedness with others				
				Connection With Others	A sense of connection to both past and future generations				
				Connection With Others	A feeling of connection with all beings				
				Connection With Others	A feeling of connection with strangers				
				Connection With Others	A sense of belonging				
				Connection With Others	A sense of connection to all living things				
4 Deconditioning and Embodiment	Aliveness	Aliveness		Aliveness	A sense of presence				
				Aliveness	Feeling totally present				
				Aliveness	A sense of still alertness				
				Aliveness	A sense of aliveness				

				Aliveness	A deep sense of being alive
				Aliveness	Feeling fully alert
6 Emotions	Unconditional peace	Equanimity		Equanimity	Feelings of equanimity
				Equanimity	A feeling of patience
				Equanimity	Experiencing unconditional peace
				Equanimity	A sense that everything is pervaded by peace
				Equanimity	An intense sense of absolute stillness and peace, even if there is chaos around me
				Equanimity	A sense of stillness and peace no matter what is happening
6 Emotions	Appreciation	Non-negativity		Nonnegativity	Experiencing few negative emotions
				Nonnegativity	Having no fear of death
				Nonnegativity	No longer being preoccupied by threats or fears
				Nonnegativity	Not feeling lonely
				Nonnegativity	Not getting angry
				Nonnegativity	No longer being concerned about other people's opinions of me
7 Foundational orientations	Unboundedness	Non-separation from everything		Non-Separation General	A sense that the boundaries of my self have dissolved
				Non-Separation General	A feeling that the boundaries between me and what is not me have dissolved
				Non-Separation General	A sense of softening of boundaries
				Non-Separation General	A sense of not being separate from my experience
				Non-Separation General	No longer feeling separate from anything
				Non-Separation General	Experiencing no distinction between me contained within the body and the world outside
4 Deconditioning and Embodiment	Aliveness	Sensitivity		Sensitivity	Feeling emotions fully
				Sensitivity	Having a rich experience of emotional territory
				Sensitivity	A capacity to notice subtle changes from moment to moment
				Sensitivity	A capacity to feel sensations of things in nature e.g., feeling the wind blowing on the leaves
				Sensitivity	Everything being perceived as vibrant
				Sensitivity	A sense of the vivid nature of ongoing experience
5 Interpersonal emotions	Open-heartedness	Unconditional love		Unconditional Love	A sense of unconditional love for everyone and everything

				Unconditional Love	A feeling of love in all my relationships
				Unconditional Love	A sense of an abundance of love
				Unconditional Love	Feelings of love
				Unconditional Love	Loving everyone as they are
				Unconditional Love	A sense of loving things as they are

Note. These are all 179 Items including those selected in EFA, classified by theme, category, hypothesized and EFA factors. Factor loadings, R^2, factor reliabilities, *means* and *standard deviations* reported for items selected in EFA. Item/Factor Loading = refers to the correlation between an observed variable (item) and a latent factor. R^2 = multiple correlation squared/measure of strength of association (square of item/factor loading); Cronbach's alpha = Cronbach's alpha reliability of multiple items; *Mean* = Average value e.g. of item scores. *SD* = Standard deviation.

Supplementary Table S3: *All 81 items and 21 factors of the Oneness Experience Scale[a]. Factor number, name, Cronbach's alpha, and oneness items in this factor*

**Factor number, name, Cronbach's[b] alpha**	**Item**
1[c] Wholeness (Part of a whole/ Connection with everything) (α=.94)	A feeling of connection to everything
	Feeling that I am part of a greater unity or oneness
	Experiencing connection with all that is
	Feeling that my individual life is part of a greater whole
	A sense of being an intrinsic part of all that is
	Feeling a sense of connection to everyone and everything
	Feeling connected with all of nature
	A sense that I am part of an all-pervading consciousness
2 Ego-dissolution (α=.95)	A feeling that my self is dissolving
	A sense of me dissolving
	A sense of dissolution of the self
	A sense of my separate identity being dissolved away
3 Clarity (α=.92)	A feeling of clarity
	A sense of clarity
	Seeing things clearly
	Everything is perceived with clarity
4 Nowness (α=.92)	Being here in the present
	A sense of being present in this moment
	Being in the moment
	A sense of being focused on the present
5 Safety and Reassurance (α=.88)	A sense of being taken care of
	A sense of safety and reassurance
	Feeling safe
	A feeling of support and love
6 Non-judgemental (α=.89)	No longer being judgemental of others
	No longer judging others behaviour
	No longer stereotyping others
	Feeling less judgemental to others
7 Timelessness (α=.90	A sense of being timeless
	A sense of timelessness
	A sense of being outside of time
	A sense of there being no time
8 Spontaneity (α=.84)	A sense of my actions happening spontaneously
	A sense of spontaneity in my actions
	My spontaneous actions feel natural
	A sense that my spontaneous actions do not need to be controlled
9 Unconditional Peace (α=.94)	Feeling tranquil
	Feelings of peace and calm
	A strong sense of peace and calm
	Feeling serene
10 Compassion (α=.84)	A feeling of great compassion
	Feelings of empathy and compassion
	Feeling great warmth towards others
	A happiness for others being happy
11 Self-compassion (α=.90)	Loving myself the way I am
	A deep acceptance and love for what I am
	Acceptance of my personality
	A feeling of self-compassion
12 Non-striving (α=.82)	No longer striving
	A sense of no longer having goals
	No longer searching for meaning
	Not forcing things to go my way

13 Spaciousness (α=.92)	A sense of limitless space<br>A sense of open, infinite space<br>A sense of being boundless<br>Experiences of boundarylessness
14 Acceptance (α=.90)	Accepting everything as it is<br>A deep sense of acceptance no matter how good or how difficult the situation<br>Allowing everything to be as it is<br>A deep sense of acceptance
15 Non-separation from others (α=.89)	A sense of not being separate from others<br>No longer feeling separate from others<br>Knowing that I am ultimately not separate from others<br>A disappearance of boundaries between self and others
16 Revelation of truth (α=.88)	Realising ultimate truths<br>A sense that the core nature of things has been revealed<br>Seeing the essential nature of reality<br>Realising who I am at the deepest level
17 Flow (α=.89)	A feeling of flow<br>A feeling of life flowing freely<br>A feeling of life flowing spontaneously<br>A sense of flow even in the face of resistance
18 Perfect as it is (α=.92)	A recognition that everything is perfect<br>Feeling that the world is perfect as it is<br>A sense that everything is already perfect and complete just as it is
19 Openness (α=.81)	Communicating openly<br>Feelings of openness<br>Being open to feel my feelings
20 Appreciation (α=.78)	Appreciating contentment and peace<br>Savouring moments of quiet and silence<br>A feeling of appreciating simple things
21 Same but different (α=.82)	A sense of being the same despite our differences<br>A sense of commonality underneath our differences<br>Feeling one with others despite differences

Note: [a] The Oneness Experience Scale begins with the following prompt:
"Please choose the answer that best describes your own opinion of what is generally true for you in your everyday life."
"My experience involves …"
and use the following Likert Scale response options: Disagree (1), Somewhat disagree (2), Neither agree nor disagree (3), Somewhat agree (4), Agree (5).
[b] The Cronbach's alpha for overall scale is .98**.** Cronbach's alphas have also been provided for individual factors, where it is desired to administer these factors on their own.
[c] Items are presented here in the order produced by the factor analysis output. To avoid possible statistical artifacts, present items in a random order during scale administration.

Citation: Van Lente, E., & Hogan, M. J. (2025). *Development and psychometric evaluation of the Oneness Experience Scale* [Manuscript submitted for publication]. *Frontiers in Psychology.*

Supplementary Table S4: *Similarities between Nondual awareness/Oneness, theoretical components, temporary mystical experience, permanent wakefulness, permanent nondual awareness, and permanent oneness scale factors*

PART 1 *Theoretical* features of permanent nondual awareness (Based on a review of Asian traditions)	PART 2 Focus is on temporary/transient mystical or enlightenment/spiritual and transformative experiences that are 'temporary' (Qualitative research)			PART 3 Focus is on permanent wakefulness (Qualitative research)	PART 4 Focus is on permanent nondual consciousness (Qualitative research)		PART 5 Focus is on permanent oneness (Qualitative and Quantitative research)	
Josipovic (2021) ("Nondual awareness")	James (1902/2002) ("Religious Experience")	Stace (1960) / Pahnke (1966) (P – Pahnke only) ("Mystical experience")	Newberg and Waldman (2017) ("enlightenment") [a] Effect – endures after experience has stopped	Kilrea, Taylor, Bilodeau, Wittmann, Linares Gutiérrez & Kübel (2023) ("Wakefulness") (Table 1)	Costeines (2009) [b] ("Nondual consciousness")	Fire (2011) [c] ("Nondual consciousness")	N	Oneness factors based on CI work and expert review Exploratory Factor Analysis Number (1-21) (Theme)
Singularity or unity// No self/self	Oneness	Oneness (2 types) 1 Extrovertive – "all things are one" 2 Introvertive Unitary Pure Consciousness /void	A sense of *unity* or connectedness	Sense of Connection and wholeness	Nondual ontology	Felt sense of No Division	1	Part of a whole/Connection with everything T7
Emptiness// No self/self		Dissolution of individuality (introvertive mysticism)					2	Ego dissolution T3
Non-conceptual reflexivity/ Luminosity or radiance		Objectivity or reality	A sense of clarity and *new understanding* in a fundamental way // Effect: more open-minded		Mental lucidity	More clarity, Felt sense of truth	3	Clarity T4
Being or Presence/ Boundless, timeless spaciousness				Increased presentness			4	Nowness T7
			Increased inner security		Awareness of spiritual immortality	More trust	5	Safety and reassurance T6
				Reduced judgemental attitude			6	Non-judgementality T4
Boundless, timeless spaciousness		Transcendence of time or space		Timelessness	Timeless awareness		7	Timelessness T7
				Authenticity, intuitive decision-making	Nondual action	Felt sense of a bigger underlying movement	8	Spontaneity T1
Bliss		Blessedness, peace / [P: deeply felt positive mood]	Effect: Happier and more contented with life	Reduced cognitive activity (mental quietness)	Beatific peace	Positively experienced	9	Peace T6
				Enhanced relationships	Positive cosmology, unitive relationships, unitive love		10	Compassion T5
					Positive cosmology		11	Self-compassion T4
Bliss (without intention)	Passivity, no control		A sense of surrender of loss of voluntary control. / Effect: Not as worried or nervous about facing and solving problems	Attitude of acceptance-letting go/surrender, changed attitude to body (not caring about one's looks)	Nondual action	More letting go and allowing	12	Non-striving T1
Boundless, timeless spaciousness		Transcendence of time or space				Non-localised, Felt sense of openness	13	Spaciousness T7
			Effect: don't ruminate on past mistakes	Attitude of acceptance/letting go/surrender		More letting go and allowing	14	Acceptance T4
Emptiness/ Nonduality	Oneness	Oneness [extrovertive]	A sense of *unity* or connectedness	Sense of connection and wholeness/ Decreased sense of group identity	Unitive relationships	Non-localised, Felt sense of mutuality, Felt sense of no division	15	Non-separation from others T7
Luminosity or radiance/ Non-conceptual reflexivity	Noetic quality (New spiritual insights)	Objectivity or reality [P: a feeling of insight or illumination]	A sense of clarity and *new understanding* in a fundamental way // Effect: more open-minded		Positive cosmology	Felt sense of truth	16	Revelation of truth T4
				Intuitive decision-making, transpersonal sense of purpose	Nondual action	More ease	17	Flow T1
Bliss					Positive cosmology		18	Perfect as it is T7
Boundless, timeless spaciousness			Effect: more open-minded	Authenticity		More letting go and allowing, Felt sense of openness	19	Openness T5
Ecstatic pleasure		Joy, happiness / [P: deeply felt positive mood]	Effect: Happier and more contented with life	Heightening of pleasure and enjoyment	Spontaneous joy, Positive cosmology	Positively experienced	20	Appreciation T6

No self/self// Emptiness/ Nonduality		Paradoxicality					21	Same and different T3
**Hypothesised factors below are not included in the final exploratory factor analysis**								
			Effect: Not as worried or nervous about facing and solving problems		Awareness of spiritual immortality / Absence of neurotic suffering		22	Non-negativity T6
Bliss		Blessedness, peace [P: deeply felt positive mood]	Effect: Happier and more contented with life		Beatific Peace, Positive Cosmology	Positively experienced	23	Equanimity T6
Emptiness/ Nonduality	Oneness	Extrovertive oneness – all things are one	A sense of *unity* or connectedness	Sense of connection and wholeness		Non-localised, Felt sense of no division	24	Non-separation (general) T7
					Unitive love, Unitive relationships, Positive Cosmology	Positively experienced	25	Unconditional Love T5
	Oneness	Extrovertive oneness – all things are one	A sense of *unity* or connectedness	Sense of connection and wholeness, Decreased sense of group identity			26	Connection with others T2
Singularity or unity// No self/self? / Boundless, timeless spaciousness	Oneness	Extrovertive oneness – all things are one	A sense of *unity* or connectedness	Nondual ontology, Sense of connection and wholeness			27	Being everything T2
Being or presence/ Non-conceptual reflexivity			An incredible intensity of experience	Increased presentness / Intensified perception			28	Aliveness T4
Ecstatic pleasure		Blessedness, peace / deeply felt positive mood	An incredible intensity of experience	Intensified perception		Energy, Dynamic	29	Sensitivity T4
Bliss/ Ecstatic pleasure		Joy, happiness[P: deeply felt positive mood]	Effect: Happier and more contented with life	Heightening of pleasure and enjoyment	Spontaneous joy	Positively experienced		Not represented : Ecstasy/joy
Non-conceptual reflexivity	Ineffability	(Alleged) Ineffability				Ineffable		Not represented: Ineffability
				Reduced identification with thoughts and mental constructs	Disidentification from mental constructs			Not represented: Disidentification

Note. [a] Newberg and Waldman (2017) also discuss a 'big E' 'Enlightenment' which is considered to be permanent. However, this has not been included in this table.
[b] The terms in this column are themes from the section *Traits of nondual consciousness* (p.116), from Costeines (2009).
[c] The terms in this column are themes from three sub-categories created in the thematic analysis, from Fire (2011): Primary category 1: Presence of nondual consciousness in one's life/ Subcategory 5: *Impact of nondual consciousness on life*, p. 146); Primary category 2: Directly felt experience of nondual consciousness itself, Subcategory 1: *Essential qualities of the felt experience of nondual consciousness* (p. 160) and Subcategory 2: *Felt Sense of Nondual Consciousness* (p. 168). "Not-represented" refer to factors covered by two or more researchers in Appendix A but not represented in the 29 oneness factors here.

References for Supplementary Materials

Costeines, M. (2009). *What enlightenment means: A qualitative study of nondual consciousness as experienced by teachers of nondual mysticism* (Doctoral dissertation). Institute of Transpersonal Psychology, Palo Alto, CA.

Fire, M. S. (2011). *Exploring the felt experience of nondual consciousness* (Doctoral dissertation). California Institute of Integral Studies, San Francisco, CA.

James, W. (2002). *The varieties of religious experience* (E. Taylor & J. Carrette, Eds.). Routledge. (Original work published 1902)

Josipovic, Z. (2021). Implicit–explicit gradient of nondual awareness or consciousness as such. *Neuroscience of Consciousness*, *2021*(2), niab031.

Kilrea, K. A., Taylor, S., Bilodeau, C., Wittmann, M., Linares Gutiérrez, D., & Kübel, S. L. (2023). Measuring an ongoing state of wakefulness: The development and validation of the inventory of secular/spiritual wakefulness (WAKE). *Journal of Humanistic Psychology*, 00221678231185891.

Newberg, A., & Waldman, M. R. (2017). *How enlightenment changes your brain: The new science of transformation*. Penguin.

Pahnke, W. N. (1966). Drugs and mysticism. *International Journal of Parapsychology*, *8*(2), 295-313.Pahnke, W. N., & Richards, W. A. (1966). Implications of LSD and experimental mysticism. *Journal of Religion and Health*, *5*(3), 175-208.

Stace, W. T. (1960). *Mysticism and philosophy*. Macmillan.
